# Sustainable Rubberized RCC Using Locally Sourced Waste Rubber Tire Powder and GGBFS-Based Binder

**DOI:** 10.3390/polym18172046

**Published:** 2026-08-23

**Authors:** İrfan Ş. Öztürk, Furkan Abdurrahman Sarı, Yakup Önal, Sercan Serin, Mehmet Emiroğlu, Hakan Güler, Tahir Gönen

**Affiliations:** 1 Department of Civil Engineering, Faculty of Engineering, Sakarya University, Sakarya 54187, Türkiye; furkansari@sakarya.edu.tr (F.A.S.); mehmetemiroglu@sakarya.edu.tr (M.E.); hguler@sakarya.edu.tr (H.G.); 2 Institute of Natural Sciences, Sakarya University, Sakarya 54187, Türkiye; 3 FAYRISE Mühendislik Teknoloji Sanayi Ticaret Limited Şirketi, Sakarya University Technology Development Zones, Sakarya 54187, Türkiye; yakup.onal@kilis.edu.tr; 4 Department of Civil Engineering, Faculty of Engineering and Architecture, Kilis 7 Aralik University, Kilis 79000, Türkiye; 5 Department of Civil Engineering, Faculty of Engineering and Natural Sciences, Osmaniye Korkut Ata University, Osmaniye 80000, Türkiye; sercanserin@osmaniye.edu.tr; 6 ZET GROUP Zöllner Eisenmann Technologie Mühendislik Sanayi ve Ticaret Limited Şirketi, Sakarya University Technology Development Zones, Sakarya 54187, Türkiye; 7 Department of Civil Engineering, Faculty of Engineering and Natural Sciences, Usak University, Usak 64200, Türkiye; tahir.gonen@usak.edu.tr

**Keywords:** waste rubber tire powder (WRTP), roller-compacted concrete, ground granulated blast-furnace slag (GGBFS), sustainable construction materials, mechanical properties, microstructural analysis

## Abstract

This study investigates roller-compacted concrete (RCC) in which fine aggregate was replaced with waste rubber tire powder (WRTP) at 0–16% by volume, using a binder containing 40% ground granulated blast-furnace slag (GGBFS). Fresh, mechanical, transport-related, microstructural, and environmental aspects were evaluated. While WRTP reduced workability, density, and ultrasonic pulse velocity (UPV), UPV values remained within the “excellent” classification range. Among the investigated mixtures, 2% WRTP provided the most favorable overall performance, increasing the 28-day compressive strength from 26.6 to 28.2 MPa, the 90-day compressive strength from 31.1 to 37.8 MPa, the flexural strength from 3.33 to 3.47 MPa, and the splitting tensile strength from 1.81 to 2.37 MPa. The 2% WRTP mixture also reduced the secondary capillary absorption coefficient by approximately 32.3%, from 0.00127 to 0.00086 mm/√s. Higher WRTP contents progressively decreased compressive strength and static modulus of elasticity, whereas flexural strength was comparatively less affected. SEM/EDS observations revealed more pronounced interfacial voids and discontinuities at higher WRTP contents, consistent with the observed performance decline. A simplified cradle-to-gate embodied-carbon assessment indicated that the 40% GGBFS binder substitution was the primary contributor to the estimated carbon reduction, with the reference and 2% WRTP mixtures exhibiting approximately 33.4% and 33.1% lower embodied carbon, respectively, than the hypothetical cement-only RCC baseline. In addition, the 2% WRTP mixture incorporated 6.43 kg/m^3^ of waste tire-derived rubber, providing an additional waste-utilization benefit. Overall, low WRTP incorporation, particularly at 2%, combined with a GGBFS-based binder provided a favorable balance between engineering performance, reduced embodied carbon, and waste utilization under the investigated conditions.

## 1. Introduction

The increase in the global population, coupled with rapid advances in industrialization and technology, has increased the demand for construction materials, resulting in the generation of large amounts of by-products and waste materials [1,2,3]. Among these, waste rubber has become a major environmental concern due to its high production volume and non-biodegradable nature [4]. While the annual production of rubber tires worldwide exceeds 3 billion units [5,6], approximately 1.5 billion reach the end of their lifespan and become waste [5]. More than half of waste rubber is stored untreated in stockpiles and landfills [7]. Waste rubber occupies vast land areas, poses a significant threat to soil and groundwater due to the toxic metals it contains, provides breeding grounds for disease-carrying pests, and increases fire risk due to its flammable composition [8,9,10]. These challenges highlight the urgent need for sustainable strategies to recycle and repurpose waste rubber [11].

One of the promising approaches is the partial or complete use of waste rubber to replace natural aggregates, which constitute the largest volume in concrete mixes and are usually derived from non-renewable resources. The use of waste rubber as aggregate offers an opportunity to conserve natural resources, prevent environmental problems caused by waste rubber, and contribute to the economy by reusing waste materials [12,13]. In this context, many studies have investigated the partial or complete replacement of natural aggregates with waste rubber in concrete production in recent years [14,15,16,17,18].

Although considerable attention has been paid in the literature to the use of waste rubber as an aggregate substitute in concrete production, the potential for using waste rubber in rigid pavements, which are gaining global popularity as an alternative to asphalt pavement due to escalating petroleum costs, has not been sufficiently investigated. Roller-compacted concrete (RCC), which is frequently used in rigid pavements, is a dry-consistency concrete preferred for airport runways, dams, parking lots, landfills, and highways. Its popularity is due to advantages such as durability, low heat of hydration, fast and easy applicability, and low cost [4,19,20]. Although RCC is produced using similar components to conventional concrete, it differs significantly from conventional concrete due to different mix proportions, lower binder (cement) content, and a higher proportion of aggregates (mostly fine-grained) [1,4,20,21]. These features make RCC a relevant platform for integrating recycled constituents, provided that compaction-driven mixture requirements are maintained.

In recent years, several studies have been conducted on the partial or complete replacement of aggregate with waste rubber powder in roller-compacted concrete pavements [4,9,22,23]. These studies generally focus on replacing fine or coarse aggregates with crushed or shredded waste rubber at various replacement levels (typically ranging from 5% to 50%). The results of these studies generally show that increasing rubber replacement ratios reduce workability and decrease mechanical properties such as compressive strength, flexural strength, splitting tensile strength, and static modulus of elasticity. However, some studies have shown that partial substitution of waste rubber up to a threshold value can provide improvements in certain mechanical properties. For instance, Hajiebrahimi et al. (2024) [22] reported an increase in flexural strength up to a 15% substitution ratio, while Fakhri and Saberi (2016) [23] reported an improvement in compressive strength up to a 10% substitution ratio and in flexural strength up to a 5% substitution ratio. Similarly, Mohammed and Adamu (2018) [9] found that compressive, splitting tensile, and flexural strength increased up to a 10% substitution ratio, with flexural strength continuing to improve up to a 20% substitution ratio. Rubber incorporation has also been investigated in RCC containing supplementary cementitious materials; for example, Mohammed et al. [24] evaluated crumb rubber in high-volume fly ash RCC incorporating nano-silica. Nevertheless, the combined use of finely graded waste rubber powder at closely spaced low-to-moderate replacement levels in a GGBFS-based RCC system remains comparatively less explored.

Overall, these findings indicate that the response of RCC to rubber incorporation is strongly dependent on rubber form, grading, and dosage, and that limited replacement levels may yield performance gains before strength and stiffness reductions become dominant at higher contents. Nevertheless, important gaps remain regarding (i) the specific use of waste rubber in fine powder form, (ii) finely spaced low-to-moderate replacement levels that enable clearer identification of a favorable performance range, and (iii) integrated evaluation across fresh behavior, mechanical–physical performance, and microstructural evidence within a single experimental framework.

Beyond aggregate replacement with waste rubber powder, the binder system provides an additional sustainability lever through supplementary cementitious materials (SCMs), particularly ground granulated blast-furnace slag (GGBFS). GGBFS has been reported to reduce the carbon footprint of concrete by lowering clinker demand and associated CO_2_ emissions [25,26], while also improving durability-related performance, such as reduced porosity/permeability and enhanced resistance to chemical attack (e.g., sulfate exposure), depending on replacement level and curing conditions [27,28,29,30,31,32]. In addition, GGBFS may offer economic and embodied-energy advantages in concrete production [26,33].

Regarding pavement-oriented applications, studies indicate that GGBFS can be effectively used in RCC/rigid pavement-related mixtures and may enhance key mechanical performance indicators (compressive, splitting tensile, and flexural strengths) [34]. Previous studies have shown that the performance of GGBFS-containing concrete is strongly dependent on the replacement level and the performance criterion considered. In RCC, Aghaeipour and Madhkhan (2017) [35] investigated GGBFS replacement levels of 0%, 20%, 40%, and 60% and reported that the 40% replacement level reduced porosity, water absorption, and permeability. The suitability of relatively high GGBFS replacement levels in RCC is also supported by Krishna Rao et al. (2016) [36], who investigated replacement levels from 10% to 60% and reported that the strength values at 7 and 28 days remained higher than those of the control mixture up to 50% GGBFS replacement. Evidence from other cementitious systems further supports the engineering relevance of this replacement level. For example, Mydin et al. (2023) [37] investigated GGBS replacement levels ranging from 0% to 60% in lightweight foamed concrete and reported improvements in compressive strength, flexural strength, splitting tensile strength, and UPV up to a replacement level of 40%. Collectively, these findings provide a literature basis for considering 40% GGBFS as a technically relevant intermediate-to-high replacement level for the present RCC system. Accordingly, a fixed GGBFS replacement level of 40% was adopted in this study to investigate the influence of WRTP within this binder system. Moreover, combining GGBFS with recycled rubber constituents has been investigated as a pathway to enhance sustainability through simultaneous waste utilization and reduced Portland-cement demand [38,39].

Despite the growing body of work on rubberized RCC, systematic studies focusing specifically on waste rubber powder (fine powder form) remain limited, and many existing studies rely on shredded/crumb rubber and standard replacement ratios (e.g., 5%, 10%, and 20%), often without evaluating fresh behavior, mechanical–physical performance, and microstructural evidence within a single integrated program. Another practical dimension highlighted in sustainability-driven concrete research is the use of locally available waste and industrial by-products to improve feasibility and reduce transportation-related impacts. In line with this approach, the waste rubber powder used in this study was obtained from a local recycling facility, and the GGBFS was sourced from local industrial production; this locally sourced character supports the practical applicability of the proposed mixtures and strengthens the circular-economy perspective of the study. In this context, the present study systematically investigates waste rubber powder as a fine aggregate substitute at finely spaced low-to-moderate replacement levels (0%, 2%, 4%, 8%, and 16% by volume) in RCC produced with a fixed binder system containing 40% GGBFS. The study adopts a multi-scale experimental program to quantify fresh consistency (Ve-Be), density, compressive strength, ultrasonic pulse velocity, flexural strength, splitting tensile strength, capillary water absorption, water absorption–porosity, static modulus of elasticity, and microstructural characteristics (SEM/EDS). Furthermore, to statistically support the empirical observations, the experimental data were evaluated using one-way, two-way, and Welch ANOVA with appropriate post-hoc comparisons (Tukey and Games-Howell, α = 0.05). In addition, age-dependent second-order polynomial regression models were used to examine the relationship between UPV and compressive strength. A simplified cradle-to-gate embodied-carbon and waste-diversion assessment was also conducted to quantitatively examine the environmental implications of the investigated GGBFS-based binder system and WRTP incorporation. Through this combined experimental, statistical, and screening-level environmental assessment, the present study aims to provide a basis for evaluating the engineering performance and environmental implications of WRTP-modified RCC within the investigated GGBFS-based binder system.

## 2. Materials and Methods

### 2.1. Materials

#### 2.1.1. Cement and Ground Granulated Blast Furnace Slag (GGBFS)

CEM I 42.5 R Portland cement conforming to TS EN 197-1 (2012) [40] was used in this study.

This study used GGBFS, a by-product of the Ereğli Iron and Steel plant and milled in the Bolu Cement plant, as a mineral admixture. GGBFS is an industrial by-product of iron and steel production and was used as a supplementary cementitious material in this study. Table 1 and Table 2 summarize the chemical composition and physical characteristics of the cement and GGBFS, respectively.

**Figure 1:**
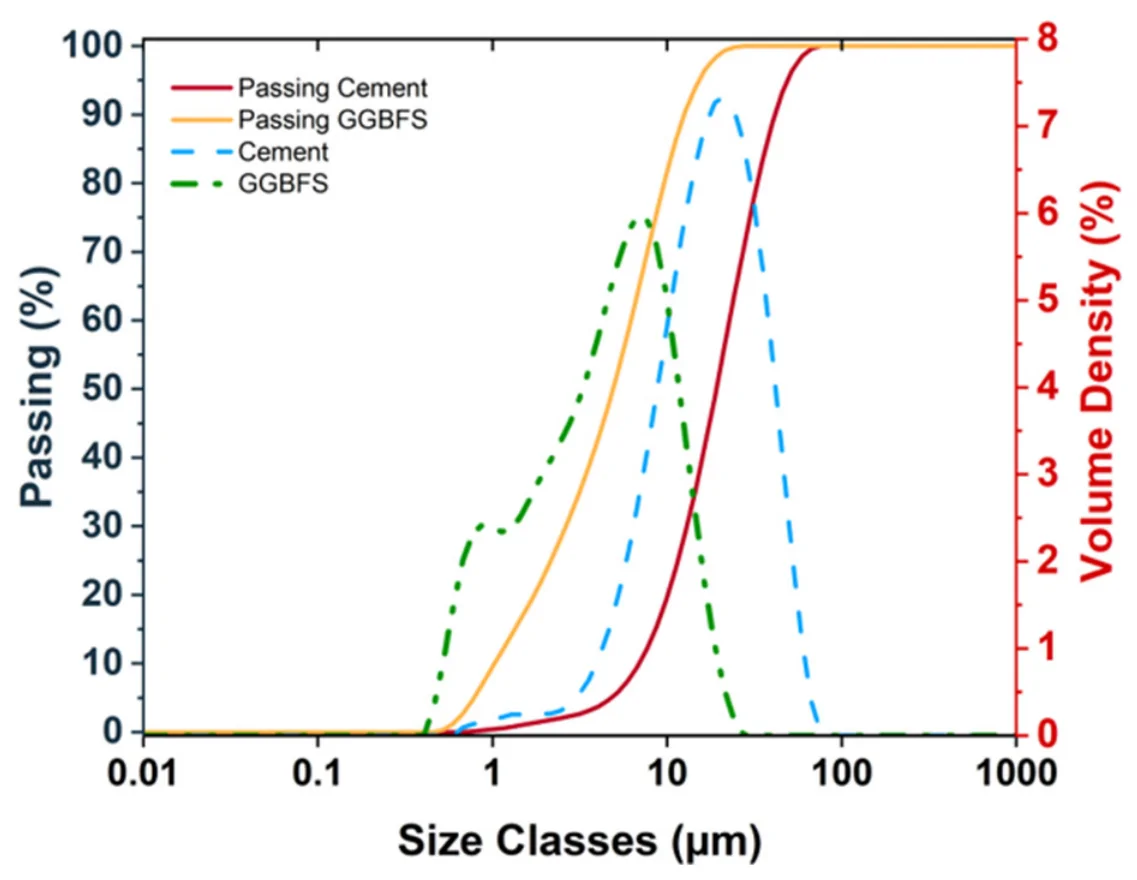
Particle size distribution and volume density plots of cement and GGBFS.

In addition, the particle size distributions of the cement and GGBFS were measured using a Malvern Mastersizer particle size analyzer, and the results are presented in Figure 1. Table 3 summarizes the D10, D50, and D90 values derived from the analysis. The homogeneity of the binder materials was characterized by calculating the Span (width) index using the values in Table 3. The Span index, calculated at 1.81 for cement and 2.35 for GGBFS, indicates that GGBFS has a finer median particle size (D50: 4.89 μm) than cement and a more broadly distributed particle size distribution. This quantitatively confirms GGBFS’s ability and efficiency to fill the voids between cement particles [41].

#### 2.1.2. Aggregate

**Figure 2:**
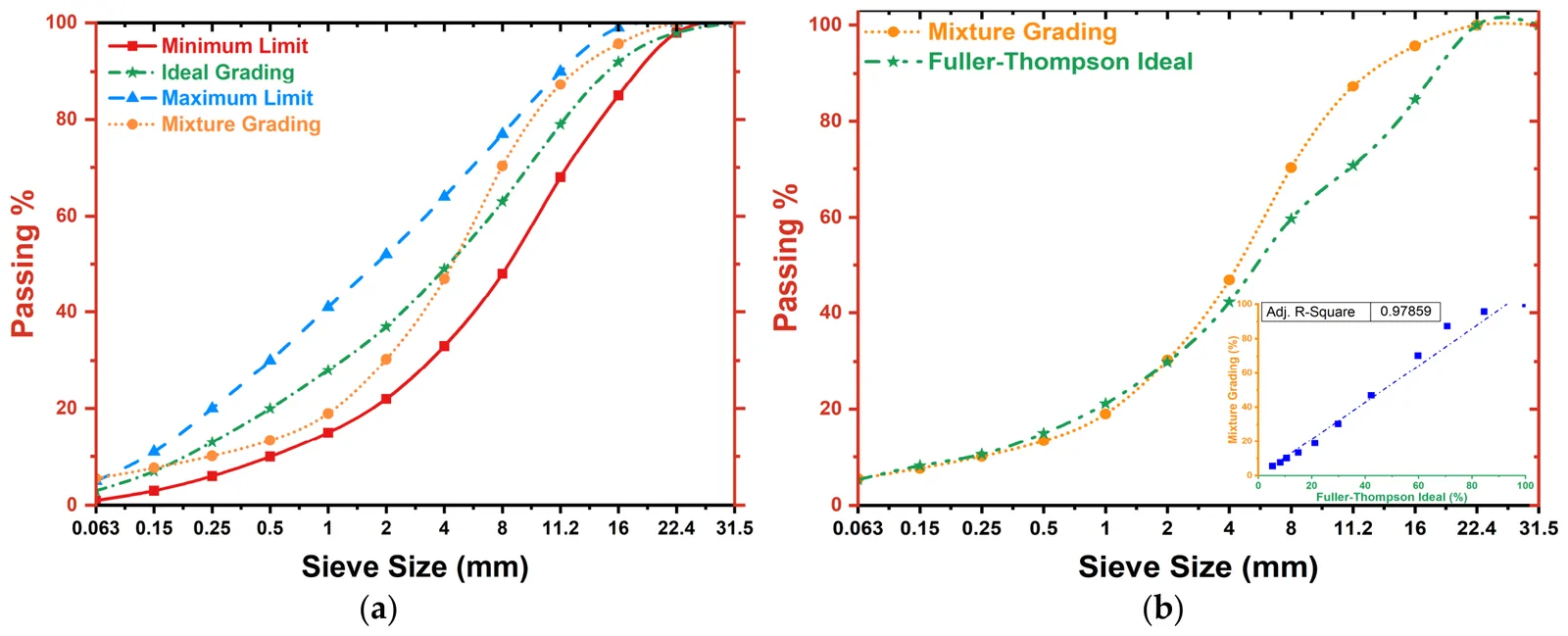
Combined aggregate gradation: (**a**) comparison with TS 802 grading limits; (**b**) comparison with the Fuller–Thompson ideal curve and regression analysis. In the inset regression plot, the blue squares represent the data points and the blue dashed line represents the linear regression fit.

The experimental study employed crushed limestone aggregates with size fractions of 0–3 mm, 0–5 mm, 5–12 mm, and 12–22 mm. Based on the particle size distributions (Figure 2), an aggregate blend comprising 4% (0–3 mm), 42% (0–5 mm), 44% (5–12 mm), and 10% (12–22 mm) by weight was selected to satisfy the grading limits recommended by TS 802 (2016) [42]. The specific gravity and water absorption capacity of the aggregates, determined according to TS EN 1097-6 (2022) [43], are presented in Table 4.

To quantitatively evaluate the aggregate gradation with respect to the maximum density and particle packing considerations adopted in the mixture design used in the study, the mixture’s gradation parameters were calculated from the gradation curve data (Figure 2). As a result of linear interpolation performed on the gradation data, the effective particle sizes were found to be D_10_ = 0.245 mm, D_30_ = 1.975 mm, and D_60_ = 6.236 mm. Based on these results, the uniformity coefficient (Cu) was calculated as 25.45 and the curvature coefficient (Cc) as 2.55. The obtained Cu and Cc values indicate favorable grading characteristics and an aggregate distribution within the optimum gradation range [44]. This mathematically supports that the aggregate gradation can contribute to reducing the voids between particles in the RCC mixture [45].

Additionally, the mixture grading was analyzed using an error analysis within the ideal median limits of TS 802, and the root mean square error (RMSE) was calculated at 5.24%, indicating that the mixture grading is close to the ideal limits. Furthermore, the regression analysis found that the mixture design’s theoretical maximum density model showed a high degree of agreement with the Fuller–Thompson curve (R^2^ = 0.98), demonstrating a strong relationship [46]. These results indicate that the selected aggregate combination provides a well-graded particle distribution, which can contribute to improved particle packing and reduced void content in RCC mixtures.

#### 2.1.3. Waste Rubber Tire Powder

**Figure 3:**
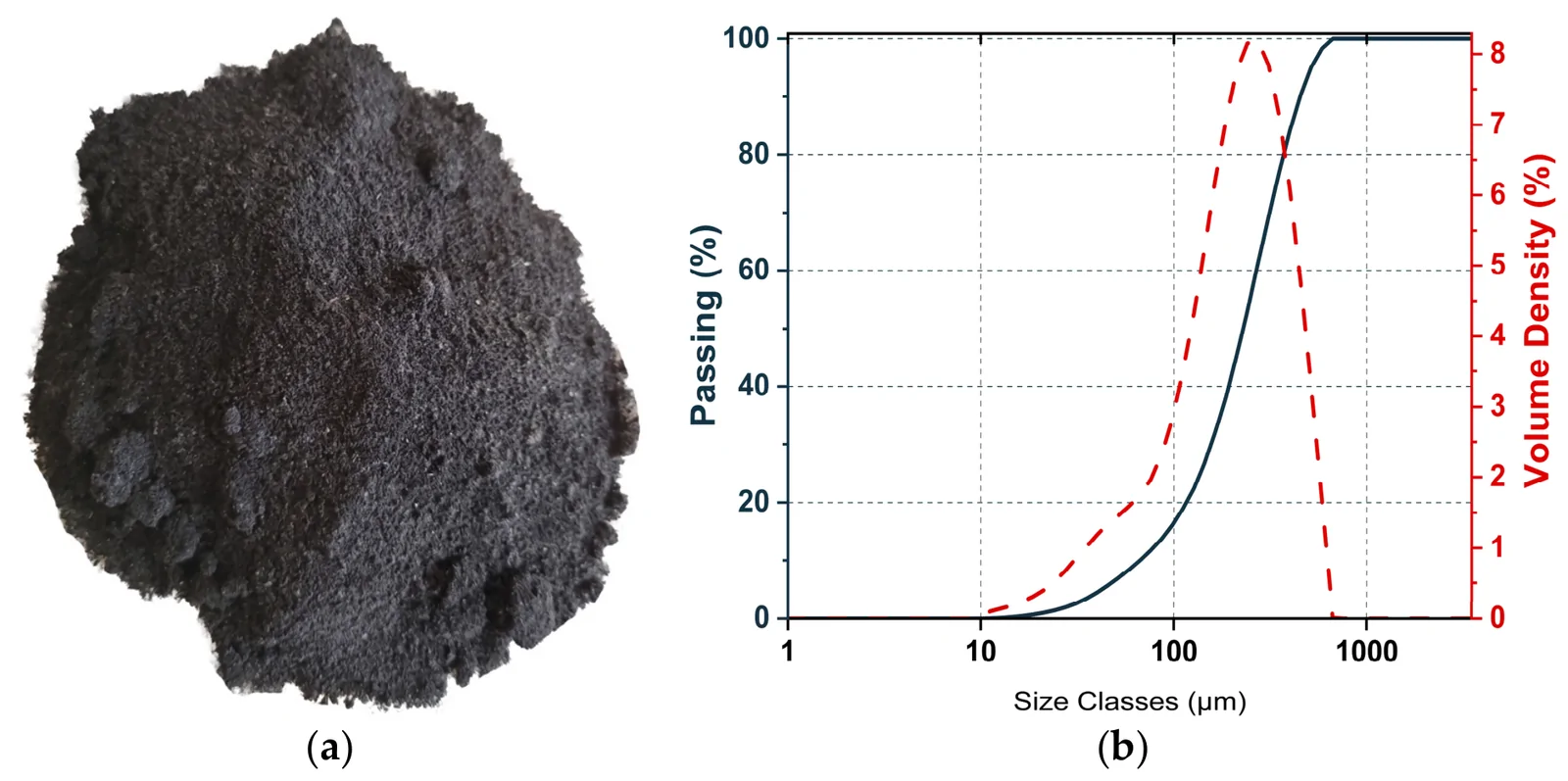
(**a**) Photograph of the locally sourced waste rubber tire powder (WRTP); (**b**) particle size distribution of WRTP, where the black solid line represents cumulative passing (%) and the red dashed line represents volume density (%).

The waste rubber powder was obtained from a local facility that recycles end-of-life tires primarily from transportation vehicles. The particle size analysis of the waste rubber powder was performed using a Malvern Mastersizer particle size analyzer; the results are presented in Figure 3. In Figure 3, particle sizes are reported in µm, and volume density is reported in %. The D10, D50, and D90 values and the specific surface area value derived from the particle size analysis are displayed in Table 5.

**Figure 4:**
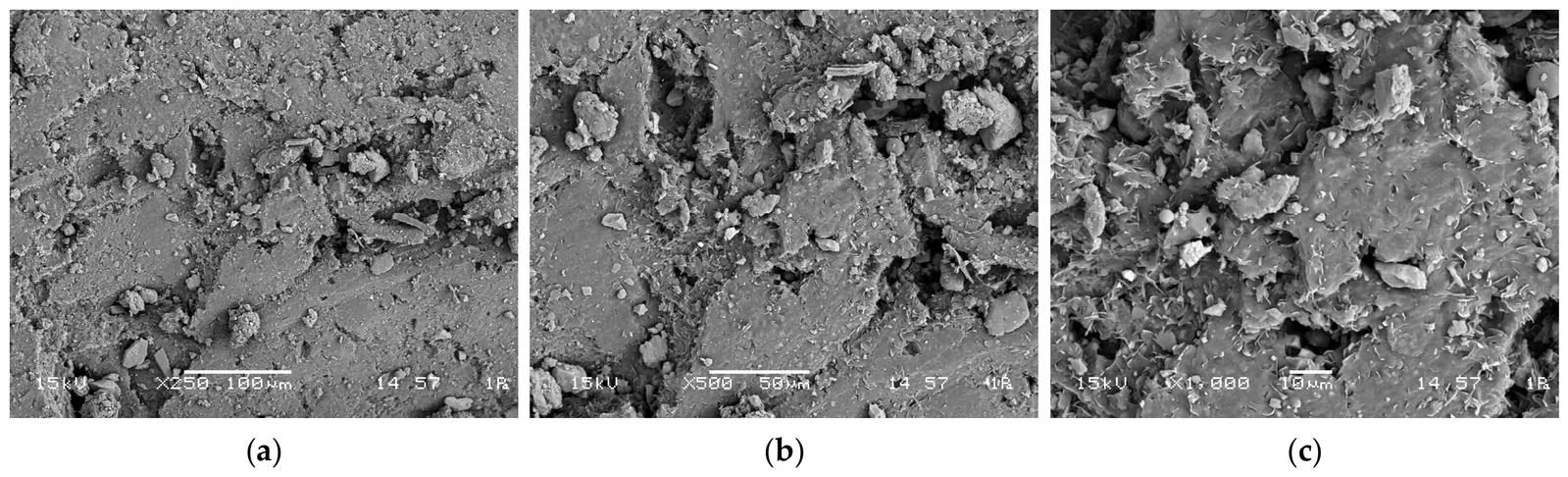
SEM images of waste rubber tire powder (WRTP) at different magnifications: (**a**) ×250, (**b**) ×500, and (**c**) ×1000.

The surface morphology of WRTP was further examined using scanning electron microscopy (SEM) at different magnifications (Figure 4). The SEM images showed an irregular and heterogeneous surface morphology with rough surface features. These observations provide qualitative information on the morphology of the WRTP particles, while their particle size distribution was determined separately by laser diffraction analysis.

Energy-dispersive X-ray spectroscopy (EDS) was also performed to characterize the local elemental composition of the WRTP. Among the detected elements, C was predominant, accounting for 49.03 weight % (65.60 atomic %), followed by O (18.83 weight %, 18.92 atomic %), Si (13.02 weight %, 7.45 atomic %), S (6.31 weight %, 3.16 atomic %) and Zn (4.56 weight %, 1.12 atomic %). Other detected elements (Ca, Al, Fe, Co, K, and Mg) were present at comparatively lower levels. As the EDS analysis represents a selected local region, these results should be interpreted as localized elemental information rather than the bulk chemical composition of the WRTP.

#### 2.1.4. Water

In this study, potable municipal water was employed for both mixing and curing. The total hardness of the water is 11.5 mg/L, the Cl value is 8.7 mg/L, the sulphate value is 10.2 mg/L, and the pH value is 7.76.

### 2.2. Mix Proportions and Mixing Procedure

Roller-compacted concrete mixes are generally designed and produced with binder contents ranging from 250 to 350 kg/m^3^, representing approximately 12% to 16% of the total dry mass of the mixture [47]. In this context, a total binder content of 300 kg/m^3^ (180 kg/m^3^ cement + 120 kg/m^3^ GGBFS) was used, corresponding to approximately 13.1% of the total dry mass of all solid constituents (300 kg/m^3^ binder and 1992.4 kg/m^3^ aggregates). When calculated relative to the total fresh mixture mass including mixing water, the binder content corresponds to approximately 12.1%. Accordingly, the binder was designed as a blended system with a GGBFS replacement level (40% of the total binder mass), which differentiates the mixture design from conventional cement-only RCC mixtures.

To determine the optimum water content, a modified Proctor-based maximum dry density approach was adopted for the RCC mixtures. Five different water contents—5%, 5.5%, 6%, 7%, and 9%—were evaluated to determine their effect on the dry density of the mixtures. The mixtures were molded in cylindrical molds measuring 150 mm in diameter and 300 mm in height using a vibrating-hammer procedure based on ASTM C1435 [48], with a modified three-layer compaction protocol [48]. To obtain a smooth surface and uniformly distribute the force produced by a vibrating hammer, a steel compaction plate was utilized. A maximum vibration time of 20 s was applied for each layer. The compacted specimens were then dried at 105 °C in an oven until a constant mass was reached. After this process, dry unit weight, wet unit weight, and water content were calculated using Equations (1)–(3):

w = [(w_wet_ − w_dry_)/w_dry_] (1)

x_wet_ = weight of wet concrete (kg)/volume of concrete (m^3^) (2)

x_dry_ = x_wet/_(1 + w) (3)

In the equations, w_wet_ and w_dry_ are the wet and oven-dry masses, respectively; w is the water content; x_wet_ is the wet unit weight; and x_dry_ is the dry unit weight.

**Figure 5:**
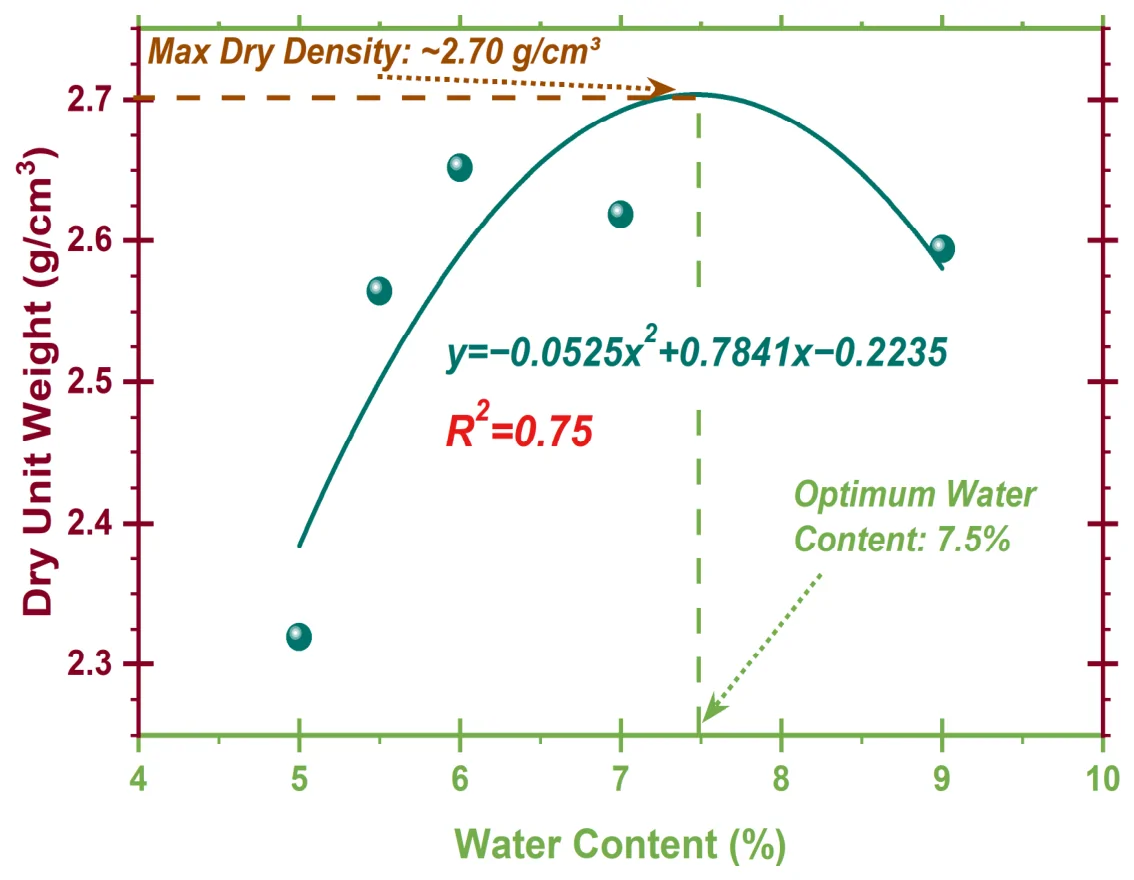
Compaction curve of the reference RCC mixture: relationship between water content and dry unit weight.

The relationship between the different water contents used in the mixtures (5%, 5.5%, 6%, 7%, and 9%) and the corresponding dry unit weight values was plotted and is presented in Figure 5. Regression-based approaches have previously been used to model compaction characteristics and determine optimum moisture content and maximum dry density [49,50,51]. Accordingly, in the present study, the experimentally obtained dry-unit-weight–water-content data were fitted using a second-order polynomial regression model to represent the compaction curve. The maximum point of the fitted curve was then determined analytically by setting the first derivative of the polynomial function equal to zero (dy/dx = 0), and the corresponding water content was identified as the optimum water content.

The calculated optimum water content was 7.47%, which was rounded to 7.5% for practical application. The optimum water content was determined using the reference RCC mixture and subsequently applied to all WRTP mixtures to ensure a consistent mixture design and enable direct comparison of the effect of WRTP incorporation. The aggregates were used in their as-received moisture condition during mixture preparation, without further adjustment of the selected water content.

Particle packing was considered during mixture design by selecting a combined aggregate gradation within the specified upper and lower grading limits for RCC. WRTP was used to volumetrically replace a portion of both fine aggregate fractions (0–3 mm and 0–5 mm), while the coarse aggregate fractions (5–12 mm and 12–22 mm) were kept constant across all mixtures. The selected WRTP replacement levels (0%, 2%, 4%, 8%, and 16% by volume) were chosen to provide a progressive range from low to moderate replacement levels, allowing the influence of increasing WRTP content on RCC performance to be systematically evaluated within the investigated range.

WRTP replacement was performed on a volume basis rather than a mass basis because the density of WRTP is considerably lower than that of natural fine aggregate. This volumetric approach allowed equivalent volumes of fine aggregate to be replaced while avoiding the disproportionate volumetric changes that would result from mass-based substitution, thereby enabling a more consistent evaluation of the influence of WRTP on the fresh and hardened properties of the RCC mixtures. Based on these mixture-design considerations and the determined optimum water content, the final proportions of all RCC mixtures were established and are presented in Table 6.

**Figure 6:**
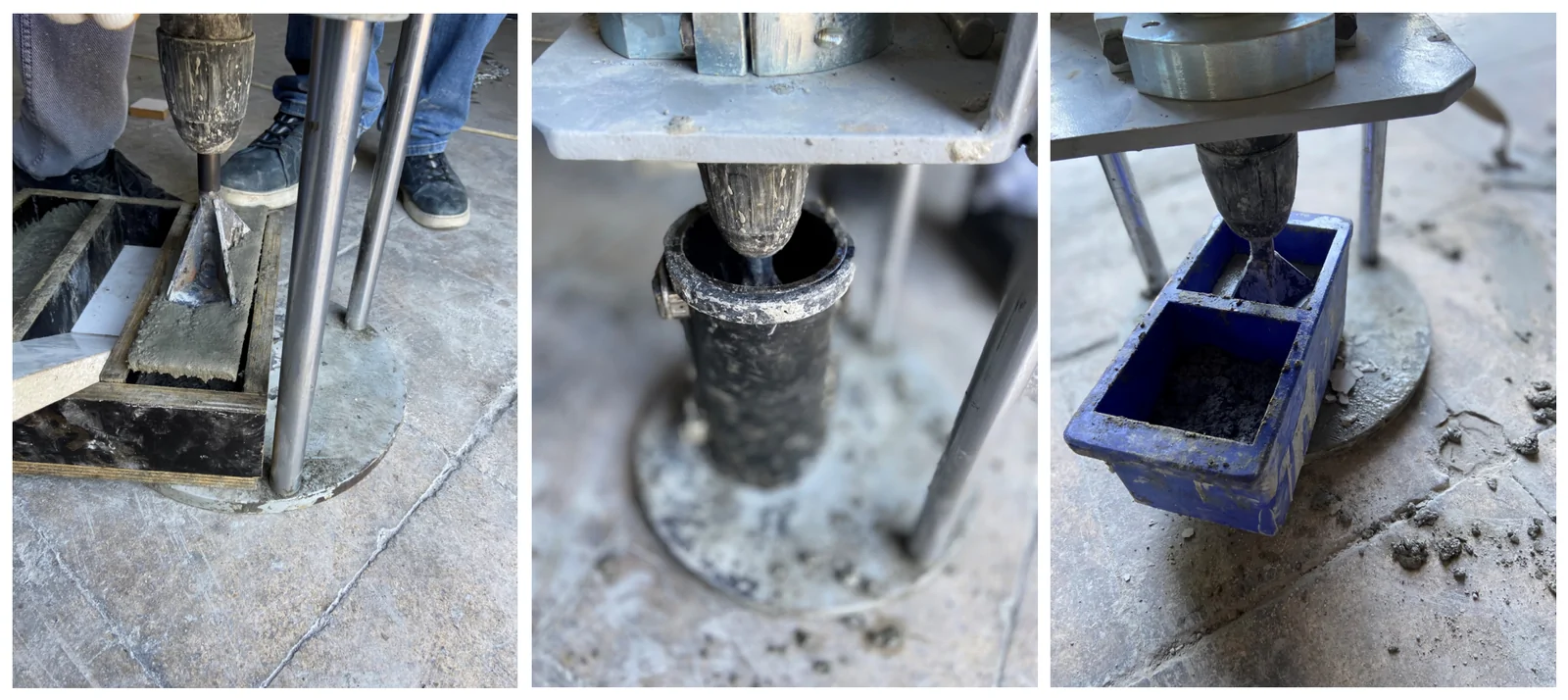
Compaction of RCC mixtures in molds using a vibrating hammer.

The mixtures prepared according to the proportions given in Table 6 were first subjected to the Ve-Be test to determine their consistency and workability. They were then placed in 100 mm cube molds, cylindrical molds (100 mm diameter × 200 mm height), and prismatic molds (75 × 75 × 275 mm) in three layers using a vibrating hammer (Figure 6). Square, rectangular and circular steel compaction plates were used to distribute the load generated by the vibrating hammer uniformly and to obtain a flat surface during compaction of each layer. A maximum vibration time of 20 s was applied to each layer. One day after casting, the specimens were demolded and cured in water until the designated testing age.

### 2.3. Test Methods and Measured Properties

The effect of the substitution of waste rubber powder at different ratios by volume (0%, 2%, 4%, 8% and 16%) instead of fine aggregate on the mechanical and physical properties of RCC was investigated using tests on fresh consistency (Ve-Be), density, ultrasonic pulse velocity (UPV), compressive strength, flexural strength, splitting tensile strength, static modulus of elasticity, capillary water absorption, and water absorption-porosity. In addition, the impact of waste rubber powder on the microstructural characteristics of RCC was examined by SEM and EDS analysis.

Statistical analyses were performed at a significance level of α = 0.05. Homogeneity of variance was assessed using Levene’s test. When the homogeneity-of-variance assumption was satisfied, one-way ANOVA followed by Tukey’s post-hoc test was applied; when this assumption was violated, Welch’s ANOVA followed by the Games–Howell post-hoc test was used. For properties evaluated at multiple curing ages, two-way ANOVA was used, where appropriate, to examine the effects of WRTP content, curing age, and their interaction. Descriptive statistics included mean values, standard deviations, coefficients of variation, and 95% confidence intervals. Statistical analyses and confidence intervals were calculated using the valid replicate measurements available for each group, with sample sizes ranging from two to four across the investigated tests and mixtures.

#### 2.3.1. Ve-Be Test

The consistency of RCC mixtures was evaluated using a Ve-Be device with a vibration frequency of 60 Hz in accordance with TS EN 12350-3 (2019) [52] standard. In this test, each mixture was placed in three layers and compacted in the slump cone inside the mold fixed on a vibrating table. Then, the slump cone was removed, the loading plate was placed slowly on the concrete, and the vibrating table was operated. The vibrating table was turned off as soon as an annular layer of mortar was formed between the interior surface of the mold and the load-applying plate. The time from the start of vibration to the formation of an annular mortar layer was recorded as the Ve-Be time. Each measurement was reported as the Ve-Be time for the corresponding mixture.

#### 2.3.2. Density

Density determination was performed according to TS EN 12390-7 (2019) [53] using three cubes (10 × 10 × 10 cm) for each mixture at 28 and 90 days. Separate specimens from the same batch were tested at each curing age. Prior to weighing, free surface water was removed from the specimens using a cloth to obtain the saturated-surface-dry (SSD) condition, following the same procedure at both curing ages. The mass (M, kg) of the specimens was then determined using a balance with appropriate precision, while the volume (V, m^3^) was determined by measuring the specimen dimensions using calipers. The density of concrete (D, kg/m^3^) was calculated as the ratio of specimen mass to volume.

#### 2.3.3. Ultrasonic Pulse Velocity (UPV) and Compressive Strength

UPV testing was performed on 10 × 10 × 10 cm cube specimens at curing ages of 7, 28 and 90 days in accordance with ASTM C597 (2016) [54] to assess the quality and uniformity of the concrete.

The UPV test measures the transit time of ultrasonic pulses through concrete specimens. The ultrasonic pulse velocity (UPV, km/s) was calculated by dividing the specimen length (L, mm) by the measured ultrasonic pulse transit time (µs).

Compressive strength tests were conducted to evaluate the effect of WRTP replacement on the compressive strength of RCC mixtures. Three cube specimens measuring 10 × 10 × 10 cm were used for compressive strength tests at 7, 28, and 90 days of age in compliance with TS EN 12390-3 (2019) [55]. TS EN 12390-3 (2019) [55] states that a loading rate between 0.4 and 0.8 MPa/s is acceptable. A loading rate of 0.6 MPa/s was used in this study. The compressive strengths of each series at 7, 28, and 90 days were calculated by averaging the results obtained from tests performed on three cube specimens.

#### 2.3.4. Splitting Tensile Strength

The splitting tensile strength test was conducted in accordance with TS EN 12390-6 (2024) [56] to evaluate the effect of WRTP incorporation on the splitting tensile strength of RCC. The loading rate was controlled to maintain a constant stress rate between 0.04 and 0.06 MPa/s. The load was applied along the casting direction of the specimen using steel plates positioned between the specimen and the upper and lower loading platens to ensure line loading and induce splitting failure along the loaded plane. The splitting tensile strength was calculated using Equation (4).

F_ct_ = [(2F)/(πLd)] (4)

In the equation, F is the maximum applied load (N), d is the specimen dimension (mm), L is the length of the line of contact between the specimen and the loading section (mm), and F_ct_ is the splitting tensile strength.

#### 2.3.5. Flexural Strength

The flexural strength test was performed on 75 × 75 × 275 mm prismatic samples according to TS EN 12390-5 (2019) [57]. The test was conducted under displacement control (at a displacement rate of 0.5 mm/min). During the tests, the span length was determined to be 225 mm, and the experiment continued until the specimen failed. The flexural strength was then calculated using Equation (5).

F_r_ = [(3PL)/(2bd^2^)] (5)

In the equation, F_r_ is the flexural strength (N/mm^2^), d is the specimen depth (mm), b is the specimen width (mm), P is the maximum load (N), and L is the span length (mm).

#### 2.3.6. Capillary Water Absorption

10 × 10 × 10 cm cube specimens were prepared for each mix series. A capillary water absorption test was conducted based on ASTM C1585 (2020) [58] with a modified specimen preconditioning procedure. For this purpose, 28-day-old specimens were dried in an oven at 105 °C until they reached a constant mass to ensure a uniform initial moisture condition for all specimens, thereby enabling a consistent comparative evaluation of the investigated mix series. The specimens were then stored under laboratory conditions until they reached a constant temperature. The specimens were then covered with waterproofing material on four sides to prevent the effect of evaporation and to ensure uniaxial water flow during testing.

Before the specimens were placed in water, their weight was measured using a balance with a 0.01 g precision. An open surface of the specimens (parallel to the casting direction) was embedded in a plastic container and exposed to water. Each specimen was placed on support rods to allow water to reach the specimen surface freely, and the water level was adjusted to approximately 4–5 mm above the specimen’s bottom. The mass of the specimens was measured at the target times specified by ASTM C1585 (2020) [58], and the mass gain at each time interval was recorded. After the experiment, capillary water absorption values were calculated using Equation 6, and a linear regression curve was plotted between capillary water absorption and time (t^0.5^). The slope of the line that best fits the capillary water absorption versus time up to 6 h is called initial water absorption, while the slope corresponding to the time from day 1 to day 7 is called secondary water absorption.

I = [(m_t_)/(a × d)] (6)

In this equation, I is capillary water absorption, m_t_ is the change in the specimen mass in grams at time t (g), d is the density of water (g/mm^3^), and a is the surface area where absorption occurs (mm^2^).

#### 2.3.7. Porosity and Water Absorption

Porosity and water absorption of the RCC specimens were determined using a modified procedure based on ASTM C642 (2021) [59], using 10 × 10 × 10 cm cube specimens cured for 28 and 90 days. Initially, the specimens were dried for 24 h at 105 ± 5 °C in an oven. The oven-dry mass of the specimens was measured using a balance with a precision of 0.01 g (w_d_). Unlike the standard ASTM C642 procedure, the boiling stage was omitted, and the specimens were immersed in water until they reached a constant saturated mass. This modified conditioning procedure was adopted to ensure identical conditioning of all specimens, thereby enabling a consistent comparative evaluation among the investigated mix series. The samples were then removed from the water, surface-dried with a cloth and weighed using a 0.01 g precision balance (w_s_). Then, the samples’ weights in water were determined using a specific gravity frame (w_w_). Porosity and water absorption values were calculated using Equations (7) and (8), respectively:

wa = [(w_s_ − w_d_)/(w_d_)] × 100 (7)

p = [(w_s_ − w_d_)/(w_s_ − w_w_)] × 100 (8)

In these equations, w_s_ is the saturated-surface-dry mass, w_w_ is the apparent mass in water, w_d_ is the oven-dry mass, wa is water absorption, and p is porosity.

#### 2.3.8. Static Modulus of Elasticity

The static modulus of elasticity of roller-compacted concrete mixtures represents the relationship between the applied uniaxial stress and the corresponding strain [60]. Cylindrical specimens with a height of 200 mm and a diameter of 100 mm were used in the test and cured for 28 days. The test was conducted using an automatic compression testing machine and extensometers to measure the deformations that occur under compressive loading. Before the start of each test, a small seating preload was applied to ensure proper contact between the specimen, the loading platens, and the extensometers. Samples from each series were tested for compressive strength prior to the static modulus of elasticity test, and the average of these values was used to determine the stress levels corresponding to 15% and 30% of the average compressive strength. The static modulus of elasticity of the samples was determined using Equation (9) by calculating the secant slope between the stress levels corresponding to 15% and 30% of the average compressive strength and their corresponding strains.

E = [(σ_2_ − σ_1_)/(ε_2_ − ε_1_)] (9)

where “E” is the static modulus of elasticity (MPa), σ_2_ and σ_1_ are the compressive stresses corresponding to 30% and 15% of the average compressive strength, respectively, ε_2_ and ε_1_ are the corresponding strains.

#### 2.3.9. Microstructure Analysis

Electron microscopy is capable of imaging objects ranging from nanometers to micrometers and is therefore used as a diagnostic instrument for micro- and nano-scale cracking in concrete. FESEM, on the other hand, has a higher magnification capability than a typical scanning electron microscope, thus offering a more advanced observation capability. In this study, the impact of waste rubber powder used as a fine aggregate substitute at different ratios by volume on the microstructural characterization of roller-compacted concrete mixtures was examined by FESEM (using an FEI Quanta FEG 450 model). The reference and 16% WRTP mixtures were selected to represent the WRTP-free matrix and the highest replacement level, respectively, while the 2% WRTP mixture was included because it exhibited the most favorable overall balance among the investigated mechanical, physical, and transport-related properties.

Material-specific elements can be characterized by using the EDS method on the surface of the samples examined by FESEM. X-rays are emitted from the specimen surface when it is bombarded with a focused electron beam. These rays detected by the EDS system provide information on the chemical composition of the material surface. In this context, the EDS method was applied to obtain qualitative information about the elemental composition of the samples produced by using waste rubber powder at different ratios by volume as a fine aggregate substitute. Accordingly, FESEM was used to observe microstructural features, while EDS was employed to qualitatively identify the elemental composition of selected areas. The SEM/EDS analyses were intended to provide qualitative and localized information on the examined microstructural features and elemental composition rather than to establish quantitative microstructural or chemical changes across all WRTP contents.

## 3. Results and Discussion

### 3.1. Ve-Be Test

**Figure 7:**
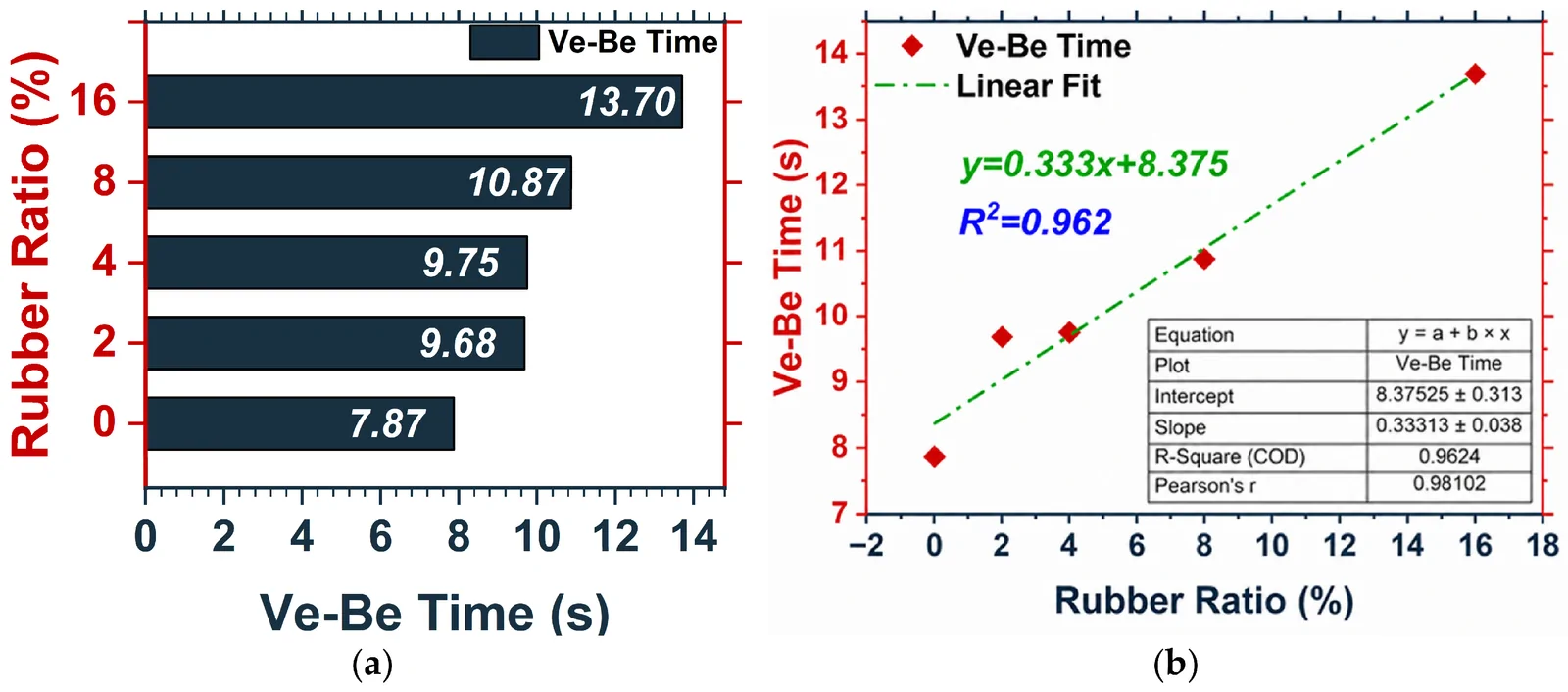
(**a**) Experimental Ve-Be times of RCC mixtures; (**b**) linear regression between WRTP content and Ve-Be time.

Figure 7 displays the findings of the Ve-Be test, which was carried out to assess the workability and consistency of RCC mixtures. Figure 7 illustrates that the Ve-Be time increases as the volume of WRTP used as a substitute for fine aggregate rises. When WRTP replaced 2%, 4%, 8%, and 16% of the fine aggregate by volume, the Ve-Be times increased by approximately 23%, 23.9%, 38.1%, and 74%, respectively, compared with the reference specimen. In terms of absolute values, the Ve-Be time increased from 7.87 s (reference) to 9.68 s, 9.75 s, 10.87 s, and 13.70 s for 2%, 4%, 8%, and 16% WRTP, respectively. This increasing trend indicates a progressive loss of workability with higher WRTP contents. Notably, this trend was observed under a constant optimum water content, indicating that the workability reduction is primarily attributable to WRTP incorporation rather than changes in mixture moisture. To statistically evaluate the effect of WRTP incorporation on fresh-state consistency, a Pearson correlation analysis was performed, yielding a strong positive correlation (Pearson’s r = 0.981, *p* < 0.01) between the increasing substitution ratio and the Ve-Be time. This result indicates a strong relationship between WRTP content and the reduction in workability. The regression-based model provided a quantitative estimation of the relationship between WRTP content and workability loss in RCC mixtures (R^2^ = 0.962). Furthermore, the horizontal trend in the Ve-Be time, which increased from 9.68 s to 9.75 s between 2% and 4%, did not cause a substantial impairment in workability compared to high replacement levels (8% and 16%). This limited increase in Ve-Be time suggests that low WRTP replacement levels (2–4%) may still provide acceptable compactability for RCC applications.

From a mechanistic perspective, the increase in Ve-Be time may be associated with the distinct physical characteristics of rubber particles relative to mineral fines. In low-slump RCC, workability is influenced by particle packing, internal friction, and the ability of the paste to lubricate contacts under vibration. The replacement of mineral fines with WRTP may reduce packing efficiency, alter interparticle friction, and promote air entrapment at particle–paste interfaces, potentially increasing the vibration time required to achieve the target remolding. These possible mechanisms are consistent with the progressive increase in Ve-Be time observed with increasing WRTP content.

Consistent with the findings of the present study, Uno and Marar (2024) [61] reported that increasing the replacement level of fine aggregate with crumb rubber (0–2.6 mm) from 0% to 40% resulted in a gradual increase in Ve-Be time. These findings suggest that incorporating waste rubber powder at increasing levels in mixtures leads to a notable reduction in the workability of RCC. Similar trends have also been reported in the literature, where increasing rubber substitution levels generally reduce workability [62,63]. Accordingly, the observed increase in Ve-Be time suggests that achieving the same level of field compaction may become more challenging at higher WRTP contents, which provides a consistent basis for the density and UPV trends discussed in the following sections.

### 3.2. Density

**Figure 8:**
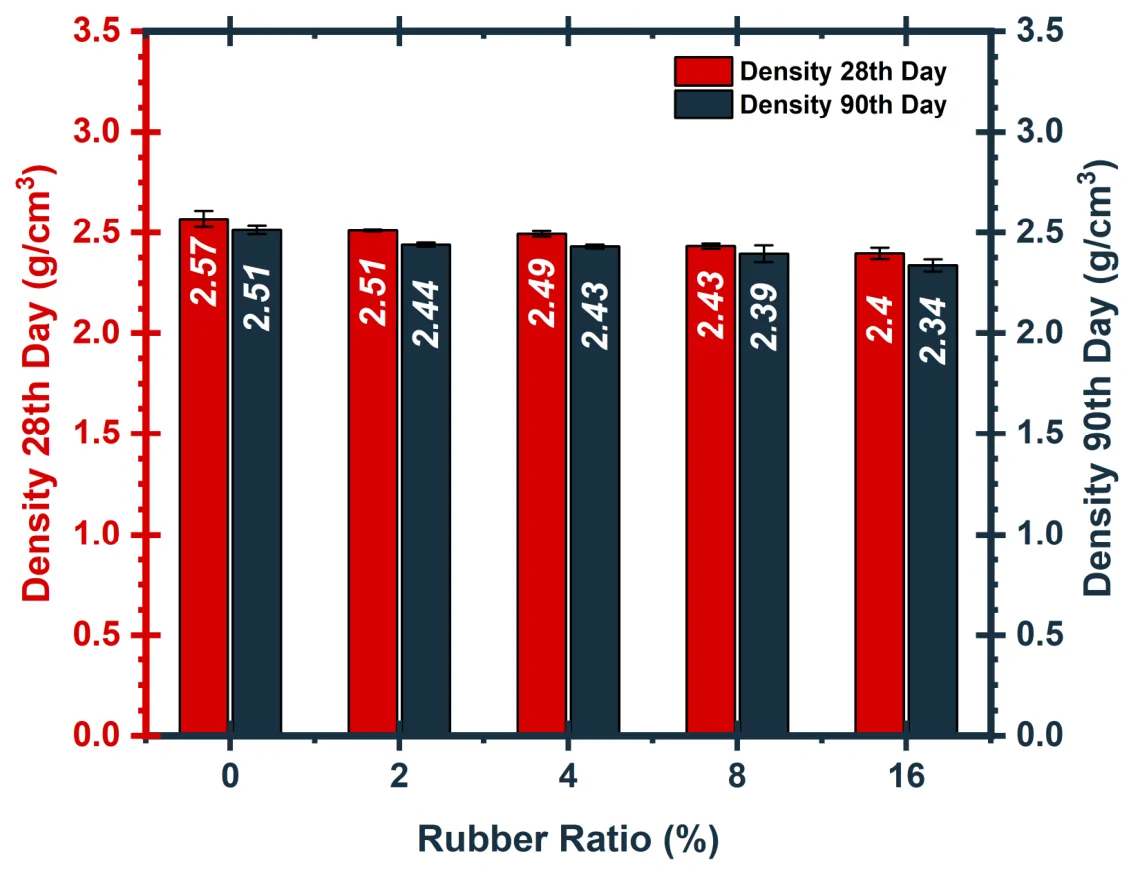
Density of RCC mixtures at 28 and 90 days.

The density values of the RCC specimens containing different WRTP replacement levels at 28 and 90 days are presented in Figure 8. Figure 8 shows that as WRTP substitution increased, the density values decreased at both 28 and 90 days. Notably, all mixtures were produced at a constant optimum water content; therefore, the density reduction observed with increasing WRTP content is primarily attributed to WRTP incorporation rather than differences in the initial mixture water content. The density values of the sample containing 16% WRTP by volume instead of fine aggregate decreased by 6.7% on the 28th day and 7% on the 90th day compared to the reference sample. For the specimens with 2%, 4%, and 8% WRTP by volume substituted for fine aggregate, the decrease was 2.3%, 3%, and 5.3% at 28 days, and 2.9%, 3.3%, and 4.7% at 90 days, respectively. In terms of absolute values, density decreased from 2.57 to 2.40 g/cm^3^ at 28 days and from 2.51 to 2.34 g/cm^3^ at 90 days as the WRTP ratio increased from 0% to 16% (Figure 8).

Although the density reduction is systematic, its magnitude remains moderate, suggesting that the compaction procedure contributed to maintaining a relatively dense skeleton even at higher WRTP contents. This observation aligns with the compaction-driven nature of RCC; nevertheless, the concurrent increase in Ve-Be time implies that achieving comparable densification becomes progressively more difficult as WRTP increases, which may contribute to the observed downward trend.

In studies where waste rubber was substituted into RCC mixtures at different ratios, it was reported that the unit weight or density values of the samples decreased with increasing waste rubber substitution ratio [4,23,64]. This decline can mainly be attributed to the lower density and specific gravity of waste rubber compared with the substituted fine aggregate. In addition to the inherently lower specific gravity of rubber, density reductions may also be associated with (i) reduced packing efficiency when deformable rubber particles replace angular mineral fines, (ii) increased air entrapment potentially related to the hydrophobic surface of rubber and less effective paste wetting, and (iii) a higher likelihood of interfacial voids at the rubber–paste interface. These possible mechanisms are consistent with the concurrent decreases in density and UPV observed in the subsequent sections. Accordingly, density can be viewed as a key intermediate parameter linking fresh workability/compaction to UPV and strength development in WRTP-modified RCC.

In addition, slight decreases in density values were generally observed from 28 to 90 days, including in the reference mixture. However, these differences should be interpreted cautiously because separate specimens from the same batch were tested at each curing age. Although the same SSD preparation procedure was applied at both ages, specimen-to-specimen variability and minor measurement uncertainty associated with the SSD condition may have contributed to the observed differences.

To rigorously evaluate the interactive and independent effects of the WRTP replacement ratio and curing age on RCC density, a two-way analysis of variance (ANOVA) was conducted, followed by a Tukey post-hoc test (α = 0.05). The statistical analysis indicated a significant effect of WRTP substitution on density (F = 42.30, *p* < 0.0001, partial η^2^ = 0.894). Post-hoc comparisons further indicated that the reduction in density became more pronounced with increasing WRTP content, particularly at the higher replacement levels. The detailed homogeneous groupings for each curing age are provided in Table A2 of Appendix A. The two-way ANOVA indicated a significant main effect associated with curing age (F = 40.17, *p* < 0.0001, partial η^2^ = 0.668), consistent with the differences in density observed between the 28- and 90-day specimen groups. More importantly, no statistically significant interaction was observed between WRTP ratio and curing age (F = 0.365, *p* = 0.8395, partial η^2^ = 0.068), indicating that the differences between the 28- and 90-day density measurements were not significantly dependent on WRTP content.

### 3.3. Ultrasonic Pulse Velocity (UPV) and Compressive Strength

**Figure 9:**
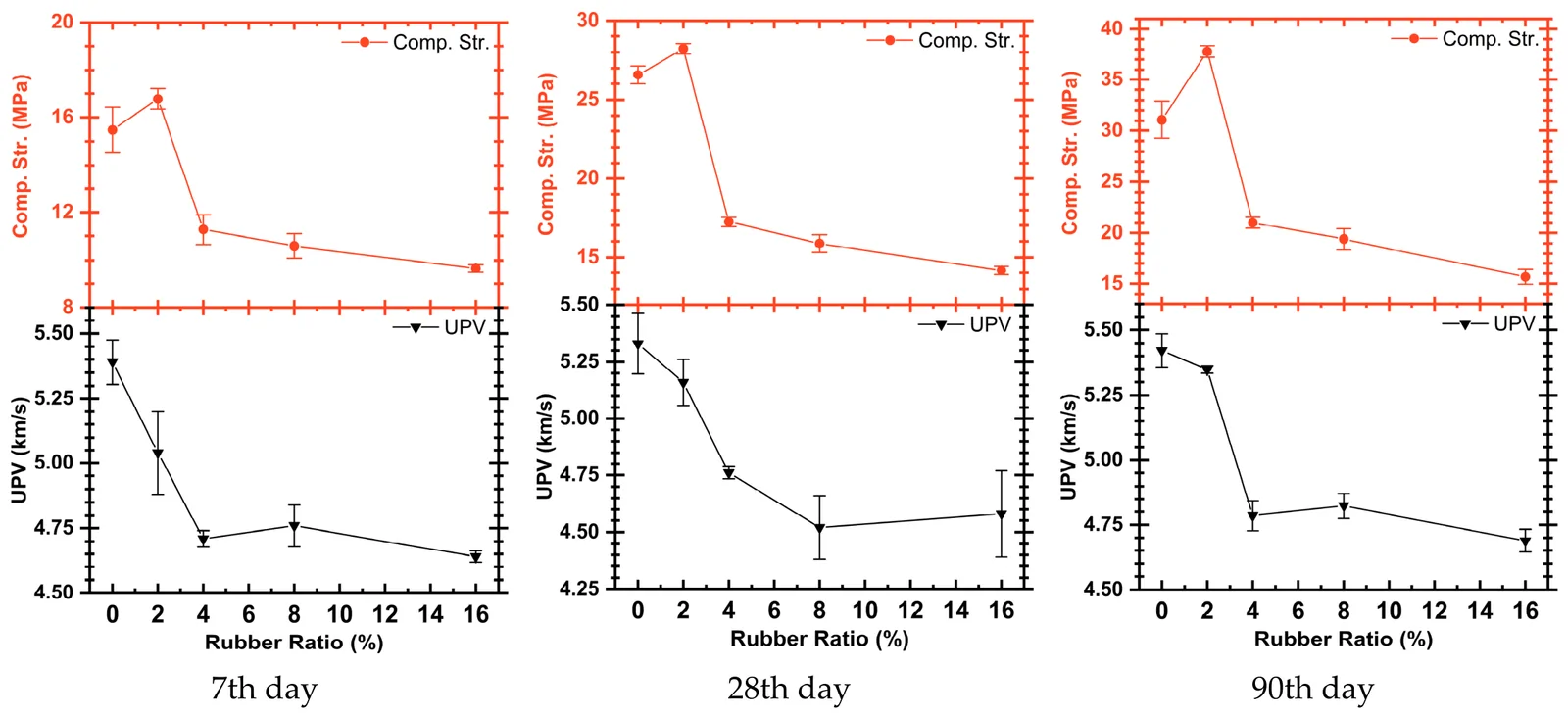
UPV and compressive strength of RCC mixtures at 7, 28, and 90 days.

The UPV and compressive strength results of the RCC specimens at 7, 28, and 90 days are presented in Figure 9. When the UPV results shown in Figure 9 are examined, the measured values range from 4.52 km/s to 5.42 km/s, and the UPV values generally increase with curing age. For the evaluation of concrete quality using UPV values, there are five quality categories [very poor, poor, doubtful, good and excellent] in the literature according to the value ranges of UPV. According to this classification, all mixtures fall within the “excellent” quality range. Compared with conventional concrete, the relatively high UPV values observed in RCC may be associated with its dry-consistency mixture characteristics and the dense internal structure achieved through compaction [65]. It should be noted that UPV primarily reflects material continuity and uniformity; therefore, it is interpreted here as an indicator of internal integrity rather than a direct measure of compressive strength.

The reference mixture exhibited the highest UPV value at all curing ages. The mixture containing 16% WRTP exhibited the lowest UPV values at 7 and 90 days, whereas the 8% WRTP mixture showed the lowest value at 28 days. The UPV values of RCC specimens containing 2%, 4%, 8%, and 16% WRTP decreased by approximately 6.5%, 12.6%, 11.7% and 13.9%, respectively, compared to the reference during the 7-day curing period. At 28 and 90 days of curing, the UPV values of the samples containing 2%, 4%, 8% and 16% WRTP by volume decreased by 3.2%, 10.7%, 15.2% and 14.1% for 28 days and 1.3%, 11.6%, 11.1% and 13.5% for 90 days, respectively, compared to the reference specimen. Overall, the reduction became more pronounced at higher WRTP contents, indicating increased discontinuities in the internal structure. The non-monotonic minimum at 28 days (8% WRTP) suggests that the UPV response may be influenced not only by the rubber volume fraction, but also by the combined effects of compaction efficiency, pore structure, and interfacial quality at a given age. In this regard, the density reductions observed in the earlier section and the interfacial voids identified by microstructural observations at higher WRTP contents provide a consistent explanation for the decrease in UPV.

Overall, the UPV values generally increased with curing age, with the 90-day values being higher than the corresponding 7-day values for all mixtures. This improvement can be associated with continued hydration reactions and progressive densification of the cementitious matrix with curing age [4]. Similar trends have been reported in previous studies, in which UPV decreased with increasing waste-rubber replacement levels in RCC [4,9,66]. Previous studies have attributed this reduction to the air entrapped around the rubber particles during mixing, which promotes the formation of a more porous concrete matrix and increases discontinuities within the internal structure [4,66,67]. This interpretation is consistent with the observed density reduction and the microstructural evidence reported for higher WRTP contents. Although all mixtures remained within the “excellent” UPV quality range, the decrease in compressive strength observed with increasing WRTP content indicates that UPV should be interpreted together with mechanical test results when evaluating the overall performance of RCC. Accordingly, the combined UPV–density trends can be interpreted as a manifestation of reduced internal continuity as WRTP content increases, even though the overall UPV classification remains within the “excellent” range.

As shown in Figure 9, the mixture containing 2% WRTP exhibited the highest compressive strength, whereas the 16% WRTP mixture exhibited the lowest value at all curing ages. The graphs show that the compressive strengths of the samples containing different ratios of WRTP by volume and the reference specimen increased with curing age. This increase is consistent with continued hydration reactions and C–S–H formation with increasing curing age [4]. In addition, as seen in the graphs, the substitution of 2% WRTP by volume improved compressive strength, whereas WRTP contents above 2% resulted in reduced compressive strength. This indicates that 2% WRTP provided the most favorable compressive performance among the investigated mixtures, whereas the strength reduction at higher WRTP contents may be associated with increased discontinuities and less effective rubber–paste interfaces.

In the literature, similar effects were observed in studies where waste rubber was substituted for aggregate at different ratios. Fakhri and Amoosoltani (2017) [68] reported a slight increase in strength at 5% waste crumb rubber replacement due to improved particle alignment and sufficient internal friction, while higher ratios (10–25%) reduced strength. Similarly, Silva et al. (2015) [69] reported a numerical increase in compressive strength at 10% recycled tire rubber replacement, which was associated with a more uniform distribution of rubber particles and improved load transfer within the matrix; however, strength declined at higher rubber contents. In this study, the fact that 2% WRTP substitution by volume also slightly increased the compressive strength in comparison to the reference may be attributed to similar effects reported in the literature. In particular, at very low replacement levels, WRTP may improve particle rearrangement during compaction and reduce stress concentrations by introducing compliant inclusions, while still preserving a continuous mineral skeleton and effective load transfer.

Compared with the reference specimens, the compressive strengths of the 2% WRTP mixture increased by 8.4%, 6.2%, and 21.6% at 7, 28, and 90 days, respectively, whereas those of the 16% WRTP mixture decreased by 37.6%, 46.7%, and 49.6%, respectively. At these curing times, the decreases in compressive strengths relative to the reference specimen were 27.2%, 35.2% and 32.4% for specimens containing 4% WRTP and 31.6%, 39.9% and 37.5% for specimens containing 8% WRTP, respectively. Notably, the severity of strength loss at higher WRTP contents is substantially greater than the corresponding reductions in flexural strength reported in the following sections, which is consistent with compressive behavior being more sensitive to matrix continuity, voids, and interfacial defects.

For RCC pavements meant for the surface layer, the American Concrete Institute recommends a minimum compressive strength of 27.6 MPa [4]. The findings of the compressive strength test within this framework indicate that the RCC mixture containing 2% WRTP by volume satisfies the compressive strength requirement for surface-layer applications. By contrast, mixtures with higher WRTP contents fall below this threshold at certain ages, which may limit their suitability for surface-layer applications unless compensated through mixture optimization (e.g., compaction energy, grading adjustments, or interface enhancement). Beyond compressive strength, the practical suitability of RCC mixtures should also be considered in relation to their fresh-state behavior and complementary engineering properties. In this respect, the relatively limited increase in Ve-Be time at low WRTP contents, together with the density and UPV results and the mechanical performance discussed in the following sections, suggests that low WRTP replacement levels, particularly 2%, can maintain a favorable overall performance balance under the investigated laboratory conditions.

The reduction in compressive strength observed at higher WRTP substitution levels may be associated with an increased thickness of the interfacial transition zone (ITZ) between the rubber powder particles and the hardened cement matrix, as well as the formation of a weaker structure associated with weak interfacial bonding [4,13,24]. It has been reported that this situation may cause loss of strength due to faster crack formation under load [4,13,24]. Previous studies have reported that increasing waste-rubber content can increase void formation and weaken interfacial bonding, thereby reducing concrete strength [4,13]. In RCC, where performance relies on achieving a dense compacted structure, such interfacial defects and entrapped voids can be particularly detrimental under compressive loading.

To evaluate the interactive effects of curing age and rubber content on mechanical performance, a two-way analysis of variance (ANOVA) was performed. The analysis indicated significant effects of curing age (*p* < 0.0001, partial η^2^ = 0.986), WRTP content (*p* < 0.0001, partial η^2^ = 0.988), and their interaction (*p* < 0.0001, partial η^2^ = 0.932) on compressive strength. The complete descriptive statistics, 95% confidence intervals, coefficients of variation, and post-hoc homogeneous groupings are presented in Table A1 of Appendix A. At 90 days, the 2% WRTP mixture exhibited the highest compressive strength (37.79 MPa), compared with 31.09 MPa for the reference mixture. These results indicate that low-level WRTP incorporation can enhance compressive performance, whereas higher replacement levels (≥4%) resulted in progressively lower strength, consistent with the increasing influence of interfacial discontinuities and voids discussed above.

**Figure 10:**
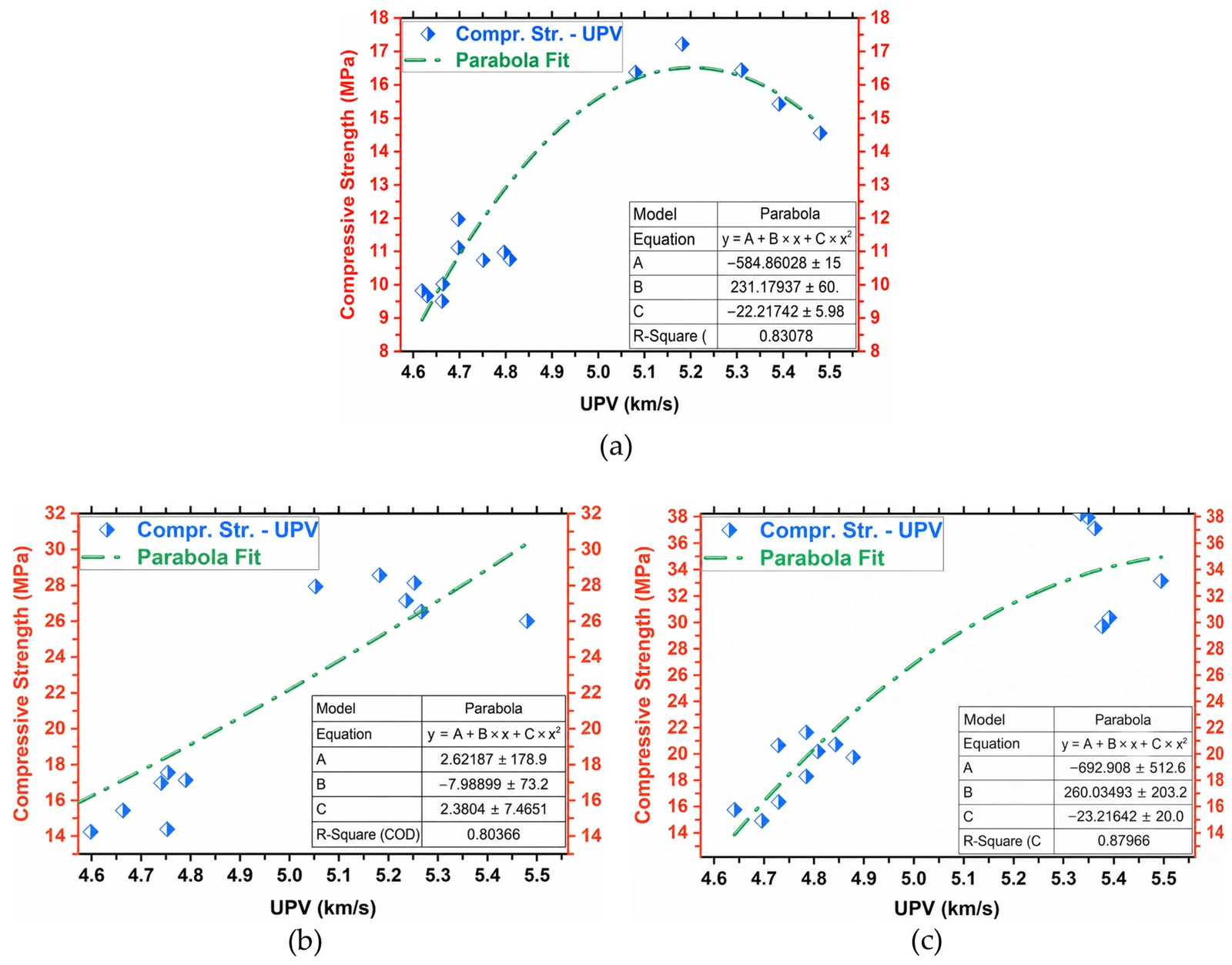
Second-order polynomial regression models relating ultrasonic pulse velocity (UPV) to compressive strength of RCC mixtures at (**a**) 7, (**b**) 28, and (**c**) 90 days of curing.

To further evaluate the relationship between ultrasonic pulse velocity (UPV) and compressive strength, age-dependent regression models were developed [70,71]. Due to the non-monotonic mechanical behavior observed at the 2% WRTP level, where compressive strength increased despite a slight decrease in UPV, a second-order polynomial (parabolic) model was adopted for each curing age (Figure 10). Model performance was evaluated using the coefficient of determination (R^2^), adjusted R^2^, root mean square error (RMSE), mean absolute error (MAE), and leave-one-out cross-validation (LOOCV), with the corresponding regression equations and performance metrics summarized in Table 7. The polynomial models yielded R^2^ values of 0.83, 0.80, and 0.88 at 7, 28, and 90 days, respectively, with corresponding RMSE values of 1.18, 2.57, and 2.85 MPa and MAE values of 0.87, 2.14, and 2.53 MPa. The LOOCV analysis yielded cross-validated Q^2^ values of 0.7642, 0.5337, and 0.8122, with corresponding CV-RMSE values of 1.39, 3.96, and 3.56 MPa, respectively.

The cross-validation results indicate that model performance varied with curing age, with stronger cross-validated performance at 7 and 90 days and comparatively lower performance at 28 days. Overall, age-dependent polynomial relationships provide useful estimates of compressive strength within the investigated dataset. However, given the limited number of observations, further validation using larger and independent datasets would be required for broader application of these relationships as NDE-based predictive models.

**Figure 11:**
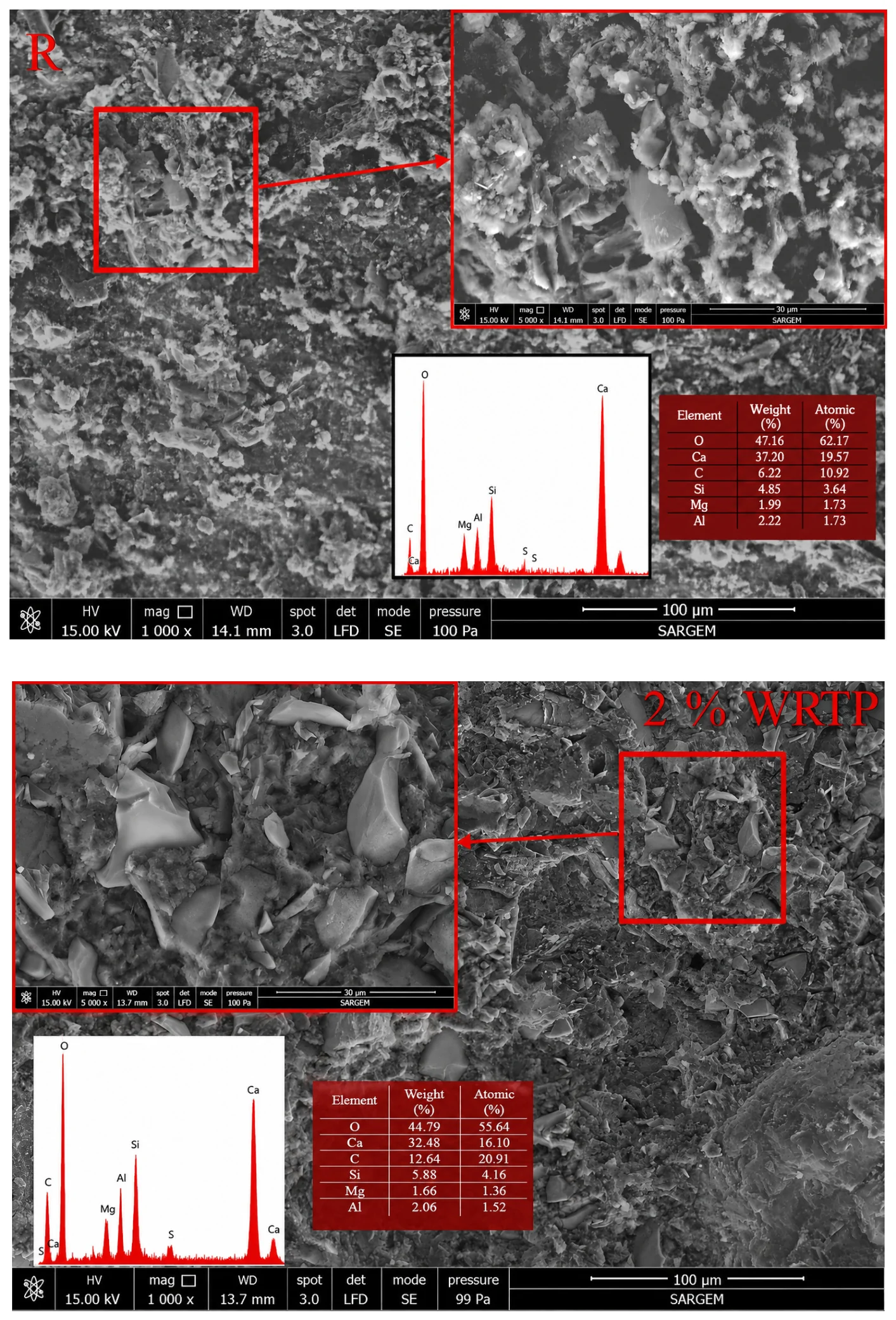

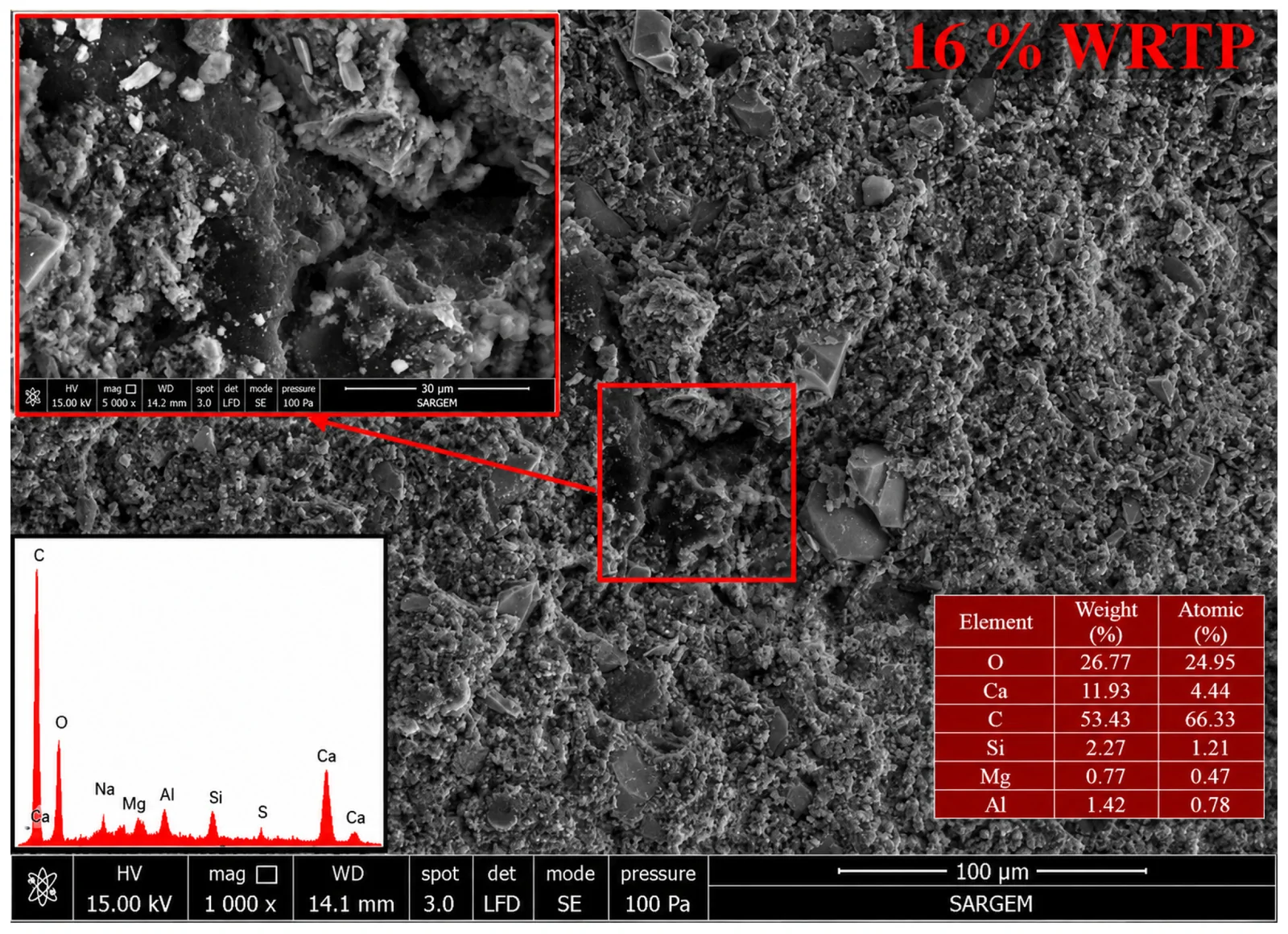
SEM images and EDS analysis of the reference, 2% WRTP, and 16% WRTP RCC mixtures.

Figure 11 presents SEM images at different magnifications of the reference, 2% WRTP, and 16% WRTP mixtures. The SEM observations reveal differences in the microstructural features of the examined regions. In the 2% WRTP mixture, the examined region exhibits a relatively continuous matrix with less pronounced interfacial discontinuities than those observed in the 16% WRTP mixture. In contrast, the 16% WRTP mixture exhibits more evident voids and discontinuities at the rubber–paste interface, suggesting less effective interfacial contact at the highest replacement level. Previous studies have similarly associated strength reductions in rubber-modified cementitious composites with weak bonding between rubber particles and the cement paste and with defects at the interfacial transition zone (ITZ) [4,9,72]. Accordingly, the more pronounced interfacial discontinuities observed in the examined regions of the 16% WRTP mixture are qualitatively consistent with its lower compressive strength, density, and UPV, whereas the comparatively less pronounced interfacial discontinuities observed in the examined regions of the 2% WRTP mixture are qualitatively consistent with its better mechanical and physical performance. As these observations are based on representative fields of view, they provide qualitative and localized microstructural support for the observed macroscopic trends rather than a quantitative characterization of the overall microstructure.

The EDS results presented in Figure 11 show differences in the carbon (C) content of the selected analysis regions among the reference, 2% WRTP, and 16% WRTP mixtures. The C content increased from 6.22 wt.% (10.92 atomic %) in the reference mixture to 12.64 wt.% (20.91 atomic %) in the 2% WRTP mixture and 53.43 wt.% (66.33 atomic %) in the 16% WRTP mixture. A similar increase in carbon content with waste rubber incorporation has also been reported in the literature [73]. The higher C contents measured locally in the WRTP-containing mixtures are consistent with the chemical characteristics of rubber-rich regions and complement the interfacial features observed in the SEM images. As the EDS measurements correspond to selected analysis regions, the observed differences reflect the local elemental composition of these regions rather than the overall distribution of WRTP throughout the RCC matrix.

### 3.4. Splitting Tensile Strength

**Figure 12:**
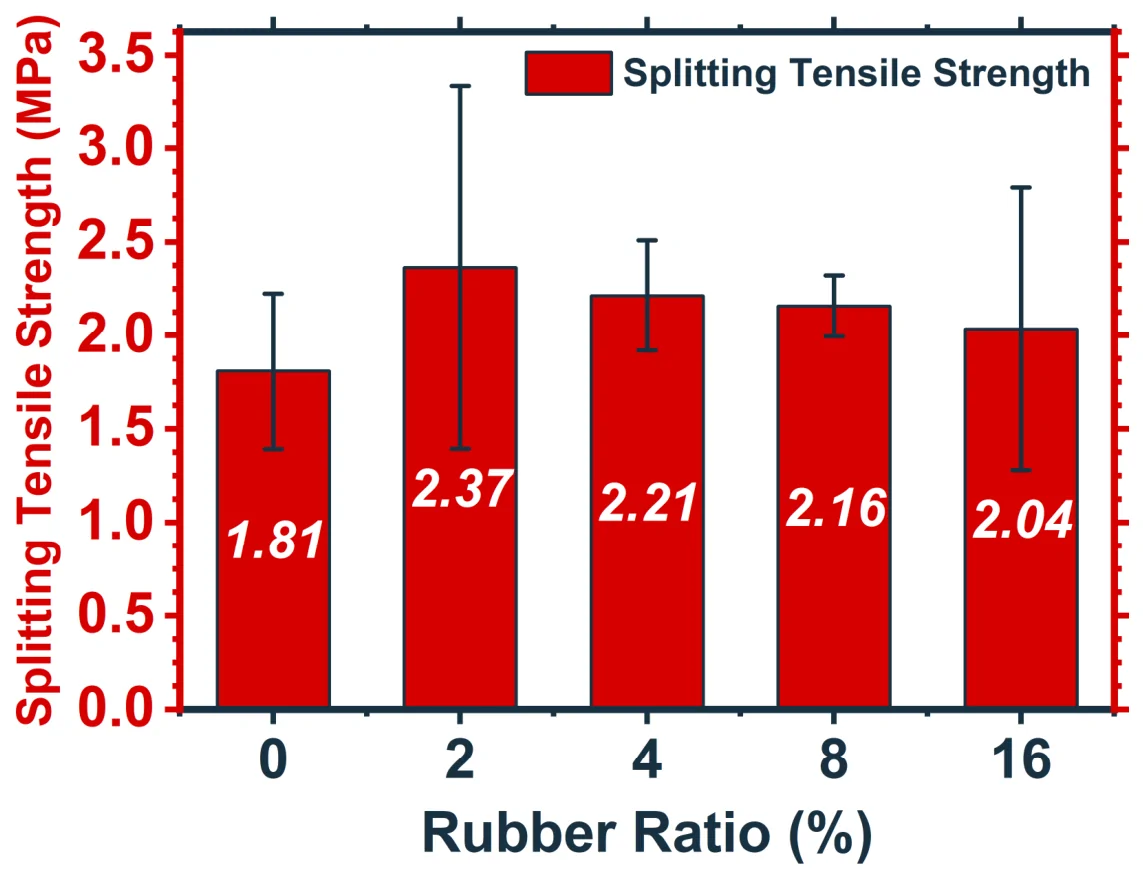
Splitting tensile strength of RCC mixtures.

The splitting tensile strength (F_ct_) values of the reference and WRTP-modified RCC specimens are presented in Figure 12.

Upon examining the values presented in Figure 12, it is evident that the F_ct_ values of the RCC samples range from 1.81 MPa to 2.37 MPa. The reference specimen exhibited the lowest (F_ct_), whereas the specimen incorporating 2% WRTP showed the highest value. As seen in the graph, the incorporation of 2%, 4%, 8%, and 16% WRTP by volume resulted in increases in (F_ct_) by 30.94%, 22.10%, 19.34%, and 12.71%, respectively, compared to the reference. In absolute terms, (F_ct_) increased from 1.81 MPa (reference) to 2.37, 2.21, 2.16, and 2.04 MPa for 2%, 4%, 8%, and 16% WRTP, respectively (Figure 12). Although the mean (F_ct_) values increased across all WRTP dosages, the magnitude of the numerical increase diminished at higher replacement levels, indicating a less pronounced positive trend with increasing WRTP content. It is also noted that the 2% WRTP mixture exhibited a wider scatter (SD = 0.97 MPa), highlighting the inherent variability of splitting tests and the sensitivity of tensile response to local defects.

Given this high standard deviation, SPSS software (version 26) was used to statistically evaluate the observed numerical differences in splitting tensile strength. Levene’s test indicated that the assumption of homogeneity of variances was violated across the mixtures, primarily due to the high variability observed in the specimens containing 2% WRTP (*p* < 0.001). Consequently, a Welch’s ANOVA was performed instead of a standard one-way ANOVA, followed by the Games-Howell post-hoc test (α = 0.05). The Welch ANOVA indicated that the WRTP substitution ratio did not have a statistically significant overall effect on the tensile strength (*p* = 0.772). Furthermore, although the mean splitting tensile strength at 2% WRTP was 30.94% higher than that of the reference mixture, the Games-Howell pairwise comparison revealed that this numerical increase was not statistically significant (*p* = 0.833). Local interfacial defects in the fracture zone and the random distribution of WRTP particles along the fracture plane may influence the failure mechanism and contribute to the characteristic high variability observed in the tensile strength of rubber-reinforced composites.

Nevertheless, the observed numerical trend may provide insight into the possible mechanisms underlying the splitting tensile response. Most studies in the literature [4,11,74,75] report a gradual decrease in (F_ct_) with increasing levels of rubber incorporation. This reduction is commonly attributed to the weak bond between rubber particles and the cement matrix, which results in microcrack development and a sudden loss of strength. In conventional (non-compacted) concretes, this weak rubber–paste interface can dominate the tensile response as rubber content increases.

Mohammed and Adamu (2018) [9] reported in their study that 10% crumb rubber substitution instead of fine aggregate increased the splitting tensile strength by 18.7% compared to the reference and that this was due to the stretching of the crumb rubber and its capability to act as a microfiber to bridge cracks. In the same study, crumb rubber substitution at higher ratios gradually reduced the (F_ct_), consistent with trends reported in the literature.

In contrast to most previous studies, the present study showed a possible tendency toward increased (F_ct_) with WRTP incorporation, as reflected by the numerically higher mean values observed across the investigated replacement levels. Compared with the reference mix, the observed numerical increases may be associated with the ability of WRTP particles to act as microfibers bridging cracks within the matrix. From a mechanistic standpoint, WRTP particles may contribute to tensile performance by promoting crack-bridging and frictional pull-out/sliding along the crack plane, which may increase energy dissipation and delay unstable crack propagation. This trend may be associated with the fine particle size of WRTP and the compaction-driven densification of RCC, which may help maintain matrix continuity and limit the adverse effects of weak rubber–paste bonding, particularly at lower WRTP dosages. Accordingly, the observed trend suggests that the compaction-driven microstructure of RCC may partially offset rubber–paste interfacial weakness at low WRTP dosages, although this interpretation requires further investigation given the statistical variability of the splitting tensile results. Notably, this tensile-strength trend is not necessarily mirrored by compressive strength, as compressive performance is more sensitive to matrix continuity and void-related discontinuities, whereas limited WRTP contents may contribute to crack resistance and energy dissipation under tensile splitting.

### 3.5. Flexural Strength

**Figure 13:**
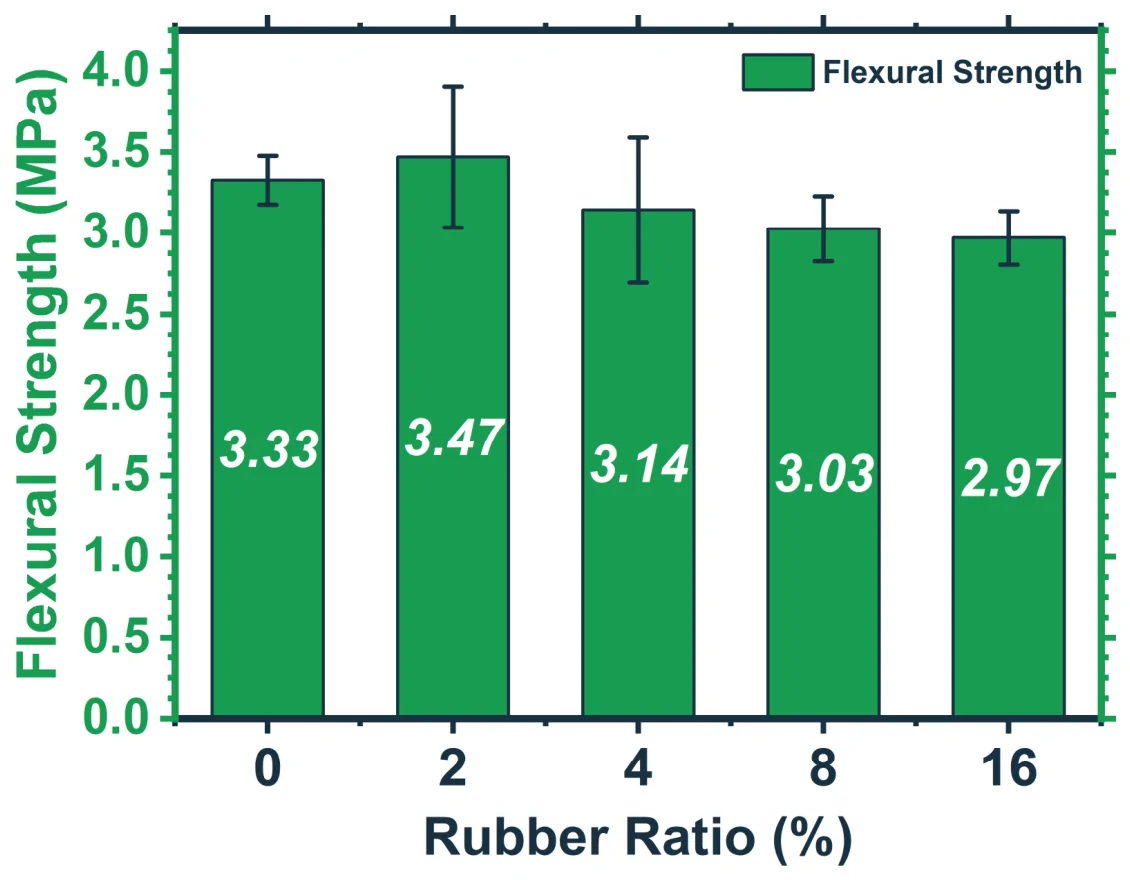
Flexural strength of RCC mixtures.

The flexural strength results of the RCC specimens are presented in Figure 13.

As shown in Figure 13, the flexural strength values of the roller-compacted specimens ranged from 2.97 to 3.47 MPa. In this study, the flexural-to-compressive strength ratio was 12–21%. This ratio is typically around 10% for conventional concrete, whereas RCC commonly shows higher values (about 12–15%) [76]. Therefore, the ratios obtained here are consistent with RCC behavior and can exceed those of conventional concrete.

The highest flexural strength was obtained for the specimen containing 2% WRTP, whereas the lowest value was recorded for the specimen containing 16% WRTP. The flexural strength value of the specimen with 2% WRTP substitution increased by 4.2% compared to the reference, while the flexural strength of the specimen with 16% WRTP substitution decreased by 10.8% compared to the reference specimen. The reductions in flexural strength relative to the reference specimen were 5.5% and 9.0% for the mixtures containing 4% and 8% WRTP, respectively. In absolute terms, flexural strength changed from 3.33 MPa (reference) to 3.47, 3.15, 3.03, and 2.97 MPa for 2%, 4%, 8%, and 16% WRTP, respectively (Figure 13). This response shows a modest numerical increase at 2% WRTP, followed by gradual reductions at higher replacement levels.

Upon examination of the results, a numerical increase in flexural strength was observed with 2% WRTP substitution by volume for fine aggregate, similar to the trend observed for compressive strength. Studies in the literature have reported increases in the flexural strength of rubber-modified concrete up to certain rubber replacement levels [9,22,23]. To statistically evaluate these differences, flexural strength was analyzed using one-way ANOVA followed by Tukey’s post-hoc test (α = 0.05). First, Levene’s test indicated homogeneity of variance across groups (*p* = 0.311), supporting the applicability of standard one-way ANOVA. The ANOVA test indicated that the WRTP ratio had no statistically significant overall effect on flexural strength (*p* = 0.327). According to the Tukey test, no statistically significant differences were observed among the mixtures, as all series, including the reference mixture, belonged to the same statistical group (Group A). This indicates that flexural strength was less sensitive to WRTP incorporation compared with compressive strength. While the 4.2% increase at 2% WRTP and the 10.8% decrease at 16% WRTP represent numerical trends, these differences were not statistically significant relative to the reference mixture. The observed flexural response may be associated with the microfiber-like behavior of WRTP particles under bending, as suggested in previous studies [4,9]. Accordingly, WRTP may contribute to post-cracking stress transfer at low dosages, whereas higher contents may promote matrix and interfacial weakening, consistent with the numerical reductions observed in flexural strength.

Mechanistically, flexural response is influenced by crack initiation and propagation in the tensile zone; therefore, limited WRTP contents may contribute to energy dissipation through crack-bridging and frictional sliding, which may help sustain load after microcracking. By contrast, compressive strength is more sensitive to matrix continuity and void-related discontinuities; thus, the adverse effects of increased interfacial defects at higher WRTP contents may be more pronounced in compression than in flexure. These observations are consistent with the density/UPV reductions and microstructural evidence of interfacial voids at high WRTP levels, which may contribute to the greater reduction observed in compressive strength compared with the more gradual numerical changes in flexural strength. From a rigid pavement perspective, this is important because flexural strength is a key design parameter for RCC surface layers. However, the observed flexural performance should be considered together with compressive strength requirements, compaction quality, and other durability and service-related properties when evaluating the potential use of WRTP-modified RCC in pavement applications.

### 3.6. Capillary Water Absorption

**Figure 14:**
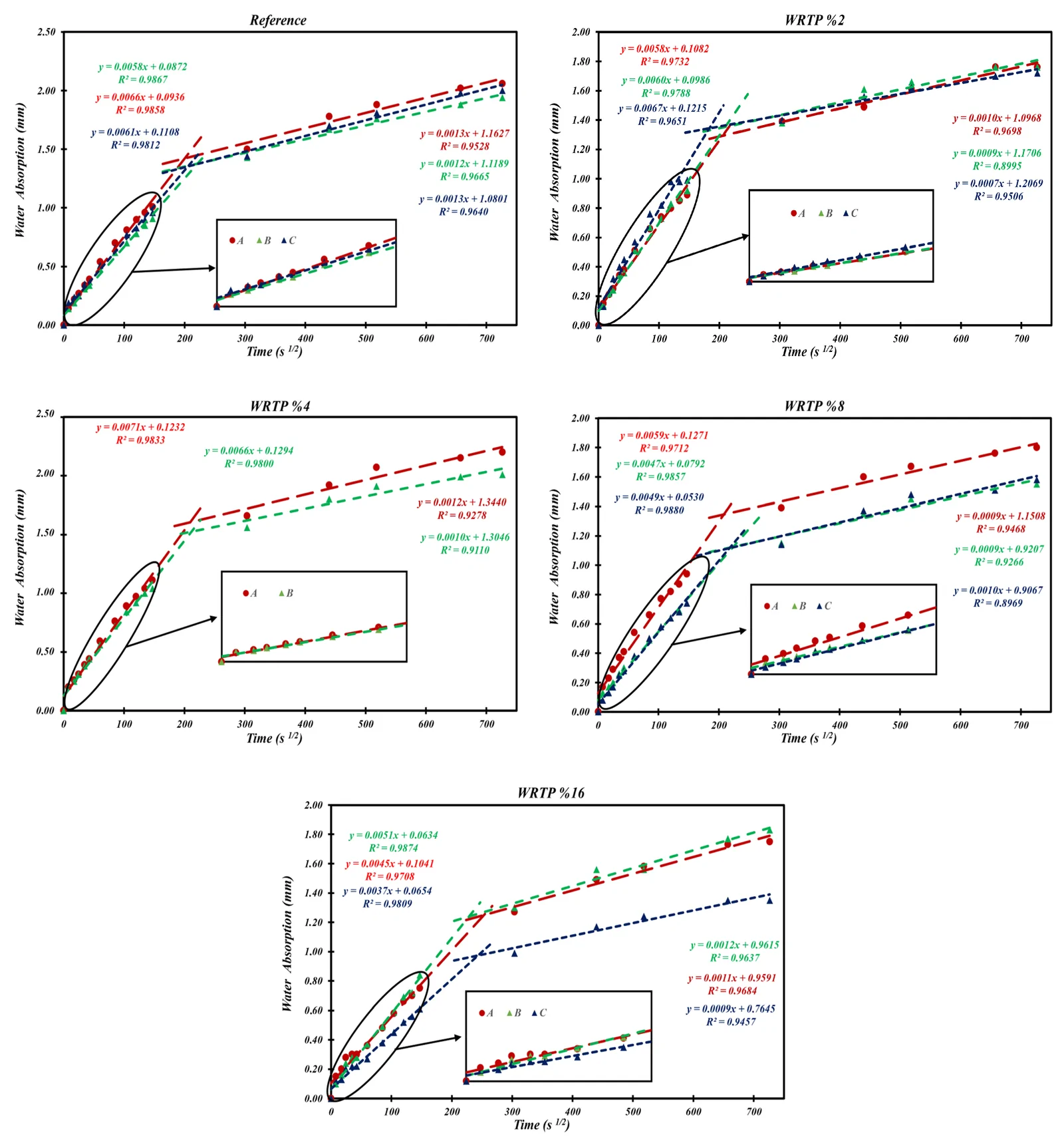
Capillary water absorption curves of RCC mixtures.

The capillary water absorption curves of the RCC specimens are presented in Figure 14, and the corresponding initial and secondary absorption coefficients are summarized in Table 8. Capillary water absorption provides useful information on water transport through the connected pore structure of concrete, which is closely associated with the durability-related transport properties of cementitious materials. In this study, capillary water absorption was therefore used to evaluate the effect of WRTP incorporation on the water-ingress behavior of RCC mixtures. In ASTM C1585-type interpretation, the initial absorption coefficient reflects the early stage of capillary water uptake, whereas the secondary absorption coefficient represents the subsequent, longer-term rate of water absorption through the connected pore network.

As shown in Table 8, the 16% WRTP mixture exhibited the lowest initial absorption coefficient, whereas the 2% WRTP mixture exhibited the lowest secondary absorption coefficient. The 4% WRTP mixture showed the highest initial absorption coefficient, whereas the reference mixture showed the highest secondary absorption coefficient. In absolute terms, the initial coefficient decreased from 0.00617 mm/√s (reference) to 0.00447 mm/√s at 16% WRTP, while the secondary coefficient decreased from 0.00127 mm/√s (reference) to 0.00086 mm/√s at 2% WRTP (Table 8).

The initial absorption values of the roller-compacted specimens containing 2%, 8%, and 16% WRTP decreased by approximately 0.5%, 16.7%, and 27.6%, respectively, whereas the initial absorption value of the 4% WRTP mixture increased by approximately 10.5% compared with the reference specimen. At higher WRTP replacement levels (≥8%), the initial absorption coefficient decreased by up to approximately 28% relative to the reference mixture. Accordingly, the initial sorptivity response was non-monotonic at low WRTP contents, with a negligible numerical change at 2% WRTP and an increase at 4%, followed by clearer reductions at higher WRTP contents (≥8%). This behavior suggests competing mechanisms: the increase observed at 4% WRTP may be associated with possible disruption of fine-particle packing and local interfacial defects, which could increase near-surface connectivity and initial uptake, whereas at higher WRTP contents, the hydrophobic nature of rubber and possible changes in pore connectivity may reduce capillary suction and disrupt continuous water pathways.

The secondary absorption values of the roller-compacted specimens with 2%, 4%, 8% and 16% replacement of WRTP showed a decrease of 32.3%, 10.3%, 26.0% and 15.8%, respectively, compared to the reference specimen. WRTP incorporation reduced the secondary absorption coefficient by up to approximately 32% relative to the reference mixture. To statistically evaluate the capillary water absorption results, a one-way ANOVA followed by Tukey’s post-hoc test was conducted (α = 0.05). The homogeneity-of-variance assumption was satisfied for both the initial and secondary absorption data (Levene’s test, *p* > 0.05). For the initial absorption data, a statistically significant difference was observed among the mixtures (*p* = 0.005, η^2^ = 0.775). According to Tukey’s post-hoc comparison, the 4% WRTP mixture was classified as Group A, the reference and 2% WRTP mixtures as Group AB, the 8% WRTP mixture as Group BC, and the 16% WRTP mixture as Group C. The 16% WRTP mixture exhibited significantly lower initial absorption than the reference and 2% WRTP mixtures (*p* = 0.026 and *p* = 0.029, respectively), while significant differences were also observed between the 4% and 8% WRTP mixtures (*p* = 0.049) and between the 4% and 16% WRTP mixtures (*p* = 0.007). These results indicate that the effect of WRTP incorporation on initial capillary absorption was non-monotonic, with statistically significant differences occurring only between selected mixtures.

For the secondary absorption data, a statistically significant difference was also observed among the mixtures (*p* = 0.018, η^2^ = 0.703). According to Tukey’s post-hoc comparison, the 2% WRTP mixture (Group B), which exhibited the lowest secondary absorption value, showed a statistically significant decrease in secondary water absorption compared with the reference mixture (Group A; *p* = 0.016). A similar reduction was also observed for the 8% WRTP mixture, which showed a statistically significant decrease compared with the reference and fell into the same subgroup as the 2% mixture (Group B; *p* = 0.0497). This statistical classification suggests that appropriate WRTP dosages can reduce secondary capillary water absorption, indicating reduced continuity of water-accessible pathways over longer absorption periods.

The consistent reduction in secondary sorptivity across WRTP dosages suggests that WRTP incorporation may reduce the long-range connectivity of water-accessible pathways, even though an increase in the initial coefficient was observed at 4% WRTP. This interpretation is compatible with the reduced UPV and density trends, which point to changes in internal continuity; however, the capillary test specifically reflects the continuity of water-accessible pathways rather than total void content.

The substitution of waste rubber may contribute to a decrease in capillary water absorption values because rubber particles can increase water repellency and may promote the formation of closed pores that are not readily accessible to water through capillary action [12,67]. Overall, WRTP incorporation tended to reduce capillary absorption across the investigated replacement levels, although the magnitude of the effect depended on WRTP content and the absorption stage. From an RCC perspective, these results imply that WRTP can reduce capillary-driven ingress provided that compaction quality is maintained, since sorptivity is highly sensitive to near-surface densification and connected porosity.

### 3.7. Porosity and Water Absorption

**Figure 15:**
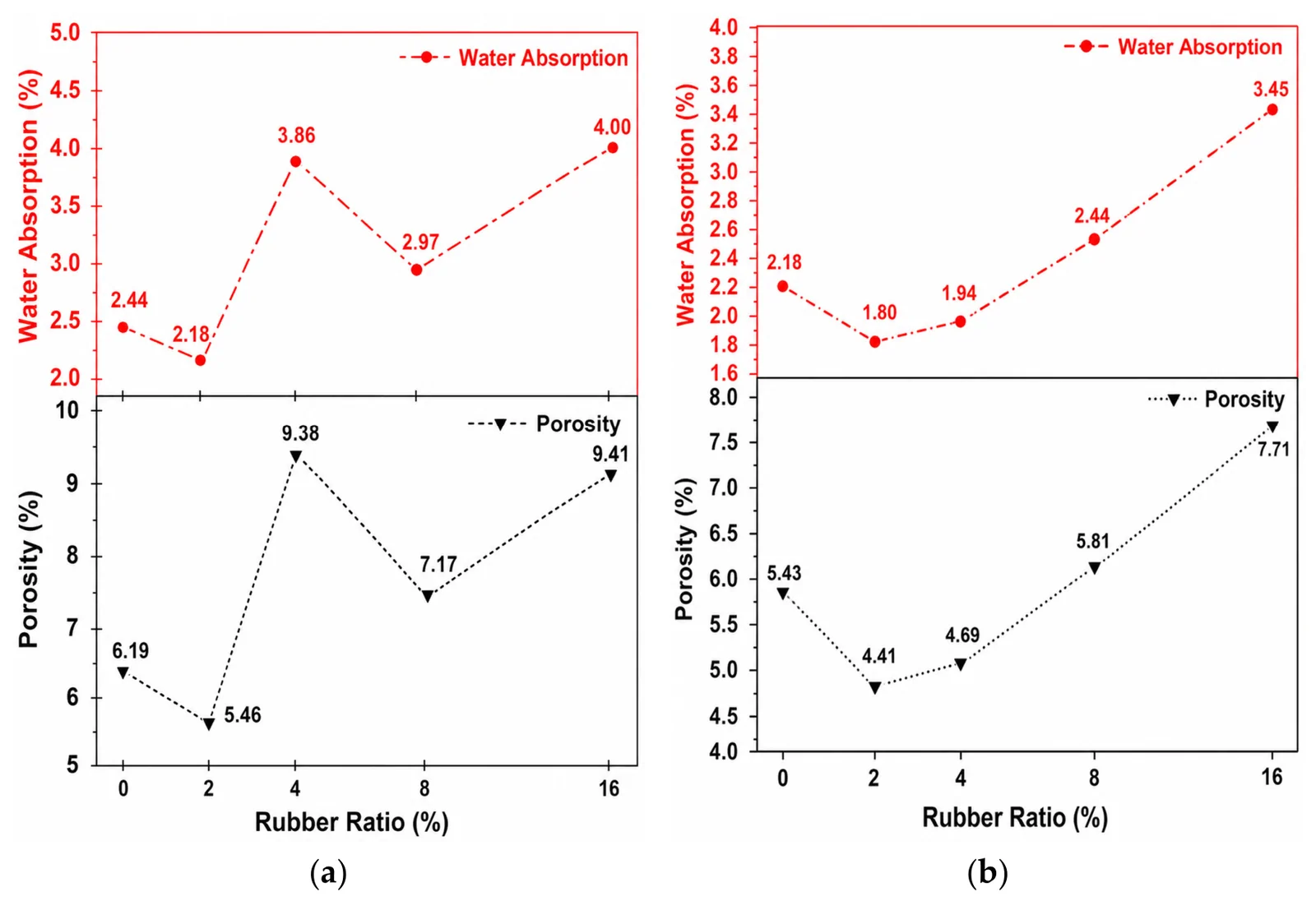
Water absorption and porosity of RCC mixtures at (**a**) 28 and (**b**) 90 days.

When examining the porosity and water absorption values of the RCC specimens on days 28 and 90 (Figure 15), it is observed that the lowest values belong to the specimen containing 2% WRTP at all curing ages. Compared to the reference sample, the 2% WRTP sample showed decreases of 11.8% and 10.8% in porosity and water absorption, respectively, on the 28th day. In contrast, the sample containing 16% WRTP exhibited an increase of 52.2% and 63.4% in porosity and water absorption, respectively, during the same curing period. Similarly, in samples containing 8% and 4% WRTP, water absorption increased by 21.55% and 58%, respectively, at the 28-day curing period, while porosity increased by 16% and 51.6%, respectively.

To statistically evaluate the 28-day water absorption and porosity results, one-way ANOVA followed by Tukey’s post-hoc test was conducted (α = 0.05). The homogeneity-of-variance assumption was satisfied for both water absorption and porosity (Levene’s test, *p* > 0.05). The ANOVA indicated a statistically significant effect of WRTP content on both 28-day water absorption (*p* < 0.0001, η^2^ = 0.978) and porosity (*p* < 0.0001, η^2^ = 0.979). According to Tukey’s post-hoc comparison for porosity, the reference and 2% WRTP mixtures were classified in Group C, the 8% WRTP mixture in Group B, and the 4% and 16% WRTP mixtures in Group A. Although the 2% WRTP mixture showed an average numerical decrease in porosity of 11.8% compared with the reference mixture, this difference was not statistically significant (*p* = 0.067).

In contrast, higher substitution levels generally resulted in significantly higher porosity, with the 8% WRTP mixture classified in Group B and the 4% and 16% WRTP mixtures in the subgroup with the highest porosity (Group A). The 16% WRTP mixture showed a statistically significant increase in porosity compared with the reference mixture (*p* < 0.0001). From a microstructural perspective, these trends suggest that WRTP may affect not only the total void ratio but also inter-pore connectivity and pore distribution. Considering that compaction is an important factor affecting the final porosity of RCC, minor changes in the quality and filling efficiency in RCC mixtures may lead to measurable differences in absorption-related indices. From this perspective, the favorable response observed at 2% WRTP may be associated with more effective compaction/filling at lower dosages. However, higher WRTP contents may promote the formation of interfacial voids, which may contribute to increased porosity and local discontinuities.

These results suggest that long-term curing and continued GGBFS hydration may have contributed to matrix densification and partially mitigated the negative effects on porosity at certain WRTP dosages through possible refinement of the connected pore structure, despite the sensitivity of rubber–paste interfaces. At 90 days, the 2% WRTP mixture exhibited decreases of 17.7% and 18.9% in water absorption and porosity, respectively, compared with the reference mixture. Similarly, the 4% WRTP mixture showed decreases of 11.0% in water absorption and 13.6% in porosity. In contrast, the 8% WRTP mixture showed increases of approximately 12.0% and 7.0%, respectively, while the 16% WRTP mixture exhibited increases of approximately 58.3% in water absorption and 42.0% in porosity relative to the reference.

For the 90-day results, the homogeneity-of-variance assumption was not satisfied for either water absorption or porosity (Levene’s test, *p* < 0.001). Therefore, Welch’s ANOVA was applied, followed by the Games–Howell post-hoc test (α = 0.05). Welch’s ANOVA indicated a statistically significant overall effect of WRTP content on both water absorption (*p* < 0.001) and porosity (*p* < 0.001). The Games–Howell comparisons showed that the statistical response varied among the WRTP levels, while the high variability observed in the 16% WRTP mixture resulted in overlapping homogeneous groups despite its higher mean values. The detailed descriptive statistics, 95% confidence intervals, and homogeneous groupings are presented in Table A2 of Appendix A. Therefore, performance should be evaluated collectively, taking into account both compaction quality (fresh stage) and pore refinement due to aging (cured stage).

**Figure 16:**
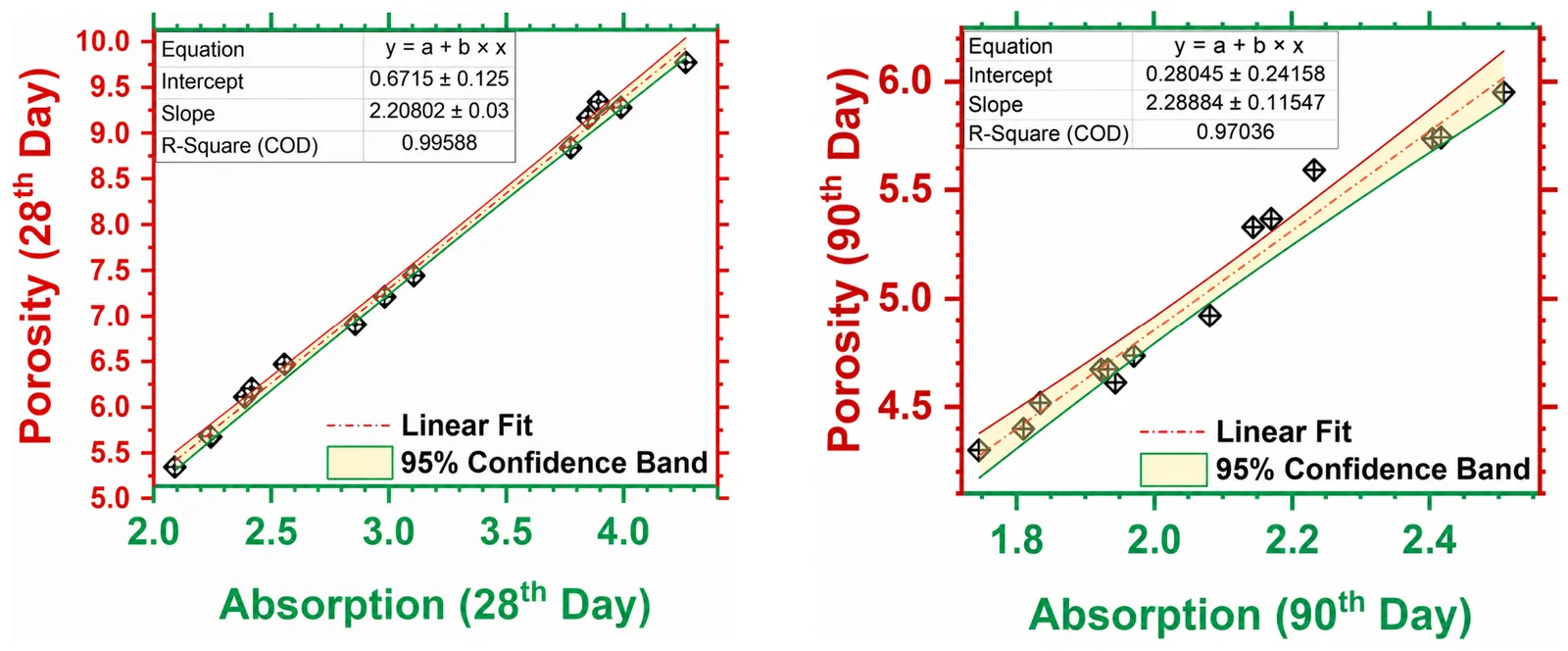
Linear relationships between porosity and water absorption of RCC mixtures at 28 and 90 days.

The water absorption capacity of concrete is closely linked to its pore structure, as concrete inherently consists of a porous material [77]. In this context, the relationship between porosity and water absorption using values at 28 and 90 days is plotted and presented in Figure 16. Figure 16 shows a strong linear relationship between porosity and water absorption at both 28 and 90 days, with R^2^ values of 0.996 and 0.970, respectively. This strong coupling indicates that changes in absorption are closely associated with the pore structure of RCC mixtures, supporting the interpretation of WRTP effects through changes in pore characteristics.

### 3.8. Static Modulus of Elasticity

When examining changes in the static modulus of elasticity values, it is observed that these values decrease as the WRTP replacement ratio increases. Accordingly, the highest static modulus of elasticity was obtained for the reference specimen, whereas the lowest value was obtained for the specimen containing 16% WRTP. The static modulus of elasticity values of the roller-compacted specimens containing 2%, 4%, 8% and 16% WRTP decreased by 2.3%, 10.4%, 16.7% and 24.9%, respectively, in comparison to the static modulus of elasticity of the reference.

In this study, a Tukey post-hoc test was performed in conjunction with ANOVA to evaluate the changes in the stiffness characteristics of the RCC matrix resulting from WRTP substitution (α = 0.05). The homogeneity-of-variance assumption was satisfied (Levene’s test, *p* > 0.05), thereby supporting the use of standard one-way ANOVA. The results indicated that WRTP substitution had a statistically significant effect on the static modulus of elasticity of the RCC mixtures (*p* < 0.001, η^2^ = 0.937). The Tukey pairwise comparisons indicated that 2% WRTP was the highest investigated replacement level that remained statistically comparable to the reference mixture. Although a 2.3% decrease in the static modulus of elasticity was noted at this 2% ratio compared to the reference sample, this change was found to be not statistically significant (*p* = 0.879), with the 2% WRTP mixture sharing the A homogeneous grouping with the reference mixture. This outcome indicates that the 2% WRTP mixture maintained a static modulus of elasticity statistically comparable to that of the reference mixture, despite the slight numerical reduction. However, the 4%, 8%, and 16% WRTP mixtures exhibited progressively lower mean static modulus values and showed statistically significant reductions relative to the reference mixture (*p* = 0.015, *p* = 0.001, and *p* < 0.001, respectively). This pronounced decrease at elevated dosages highlights the influence of higher WRTP contents on internal stiffness and may also reflect changes in interfacial bonding.

In absolute terms, the static modulus of elasticity decreased from 13.65 GPa (reference) to 13.34, 12.23, 11.37, and 10.25 GPa for 2%, 4%, 8%, and 16% WRTP, respectively. This monotonic reduction indicates a stiffness-controlled response typical of rubberized cementitious composites, where compliant inclusions reduce the static modulus of elasticity even when strength changes are modest at low dosages. Beyond the intrinsic low modulus of rubber, the reduction may also be associated with weaker rubber–paste bonding and interfacial voids, which may reduce the efficiency of stress transfer and increase deformability under the same stress level. Accordingly, the observed trend in the static modulus of elasticity is consistent with the reductions in density and UPV as well as the microstructural evidence of interfacial discontinuities at higher WRTP contents.

These findings are consistent with results reported in the literature. For example, Hajiebrahimi et al. (2024) [22] investigated the effect of crumb rubber aggregate substitution at various levels (0%, 10%, 15%, 20%, 35%, and 50%) and found a clear reduction in the static modulus of elasticity with increasing rubber content. Similarly, Meddah et al. (2014) [10] reported a decreasing trend in the static modulus of elasticity of roller-compacted concrete pavements as the substitution rate of rubber particles increased (0% to 30% by volume). Gupta et al. (2016) [78] also observed a decline in the static modulus of elasticity in concretes incorporating rubber fibers at replacement levels ranging from 0% to 25%, produced with various water/cement ratios.

The lower static modulus of elasticity of rubber-substituted concrete compared to the reference concrete can be attributed to the lower static modulus of elasticity of rubber particles in comparison to the substituted aggregate and the weak adhesion between the cement matrix and the rubber aggregate [16,78,79]. In RCC applications, this stiffness reduction may contribute to improved energy absorption and crack tolerance, while also potentially affecting pavement deflection behavior; therefore, WRTP dosage should be selected by considering both strength and stiffness requirements.

**Figure 17:**
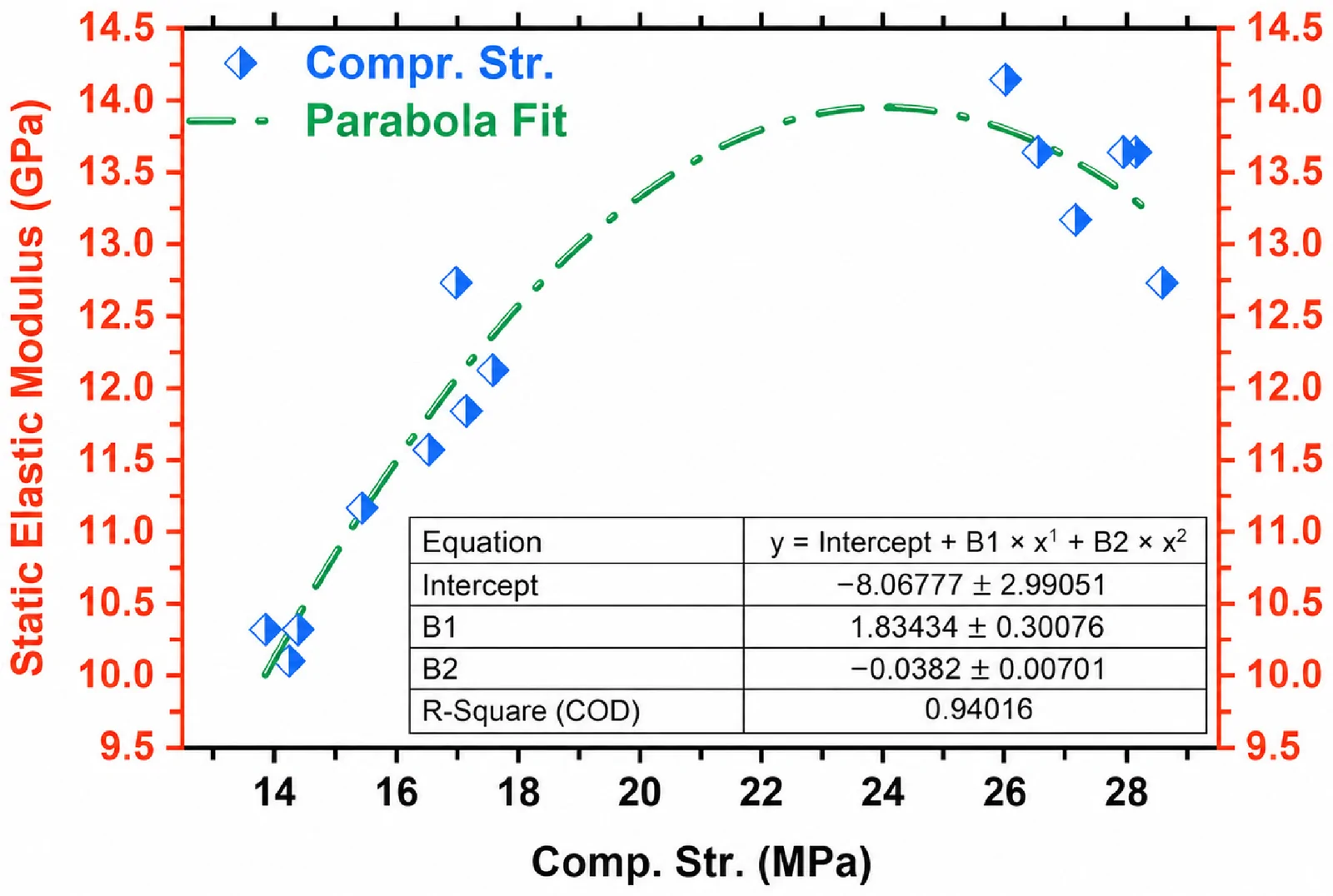
Second-order polynomial relationship between 28-day compressive strength and static modulus of elasticity of RCC mixtures.

To gain a more detailed understanding of the mechanical behavior, the relationship between the static modulus of elasticity and the compressive strength of RCC specimens was examined. A strong positive correlation was observed between compressive strength and static modulus of elasticity. A polynomial regression analysis was performed to empirically describe this relationship, shown in Figure 17.

A high coefficient of determination (R^2^ = 0.94) was obtained, indicating a strong relationship between compressive strength and static modulus of elasticity within the investigated RCC mixtures. The nonlinear regression model provides an empirical description of the relationship within the range of mixtures investigated in this study and should not be interpreted as a general predictive model for rubberized RCC. It was observed that WRTP replacement levels above 2% simultaneously reduced both the static modulus of elasticity and compressive strength, indicating that the lower stiffness of rubber particles and the presence of interfacial voids may contribute to reduced stiffness and strength at higher replacement levels.

### 3.9. Simplified Embodied Carbon and Waste Diversion Assessment

To provide a quantitative screening-level assessment of the environmental implications of the investigated roller-compacted concrete (RCC) mixtures, a simplified cradle-to-gate embodied carbon and waste-diversion analysis was conducted. Reducing clinker consumption through the incorporation of supplementary cementitious materials (SCMs) is widely recognized as an important strategy for lowering the embodied carbon of cementitious materials [80], while life-cycle-based approaches provide a useful framework for evaluating the environmental impacts of alternative concrete mixtures [81]. In addition, the utilization of industrial by-products and recycled waste materials can contribute to resource efficiency and waste valorization in cementitious systems [82].

The embodied carbon of each mixture was estimated from the mass of its constituent materials and their corresponding embodied carbon factors (ECFs) according to:

(10) $${\mathrm{EC}}_{\mathrm{mix}}=\sum_{i=1}^{n}(M_{i}\times ECF_{i})$$

where EC_mix_ is the estimated embodied carbon of the mixture (kg CO_2_-eq/m^3^), M_i_ is the mass of constituent i per cubic meter of RCC (kg/m^3^), and ECF_i_ is the corresponding embodied carbon factor (kg CO_2_-eq/kg).

The ECFs adopted in the present assessment were obtained from peer-reviewed literature. Liu et al. (2024) [83] reported ECFs of 0.735 kg CO_2_-eq/kg for cement, 0.0901 kg CO_2_-eq/kg for GGBFS, 0.0039 kg CO_2_-eq/kg for coarse aggregate, and 0.0002 kg CO_2_-eq/kg for water. For the 0–3 mm and 0–5 mm crushed fine aggregate fractions used in this study, an ECF of 0.007651 kg CO_2_-eq/kg was adopted as a literature-based proxy from the manufactured fine aggregate value reported by Zhu et al. [84]. The same ECF was also reported by Liu et al. [83]. The use of literature-derived ECFs enables a consistent comparative assessment of the selected mixtures, although absolute values may vary depending on material source, production technology, regional energy mix, allocation procedures, and system boundaries.

Because a product-specific Environmental Product Declaration (EPD) or process-specific life-cycle inventory was unavailable for the locally sourced WRTP used in this study, its ECF was represented using a literature-based proxy for mechanically recycled tire rubber. Sudan et al. (2025) [85] conducted a cradle-to-gate life-cycle assessment of end-of-life tire recycling into crumb rubber (CR), micronized rubber powder (MRP), and reclaimed rubber. For CR produced from domestic end-of-life tires, a global warming potential of 1.13 × 10^4^ kg CO_2_-eq per 100 metric tons of final product was reported, corresponding to 0.113 kg CO_2_-eq/kg. This value was therefore adopted as the WRTP proxy in the present screening-level assessment. The use of recycled crumb rubber as a literature proxy is further supported by the independent life-cycle assessment of tire-to-crumb-rubber recycling reported by Tushar et al. (2022) [86]. Nevertheless, because the adopted factor does not originate from the specific local WRTP production facility used in the present study, it should be regarded as a literature-based screening value rather than a product-specific carbon footprint.

For comparative purposes, the assessment considered the production of 1 m^3^ of three selected mixtures: a hypothetical cement-only RCC baseline, used solely for the screening-level environmental comparison, containing 300 kg/m^3^ of cement; the reference RCC mixture (R) containing 40% GGBFS as a binder replacement; and the 2% WRTP mixture containing 40% GGBFS and 2% WRTP as a volumetric fine-aggregate replacement. The hypothetical cement-only mixture was not experimentally produced or tested and was included solely as a comparative baseline for evaluating the estimated embodied-carbon effect of the 40% GGBFS binder substitution. The constituent quantities, adopted ECFs, estimated embodied carbon, and WRTP utilization are summarized in Table 9.

**Figure 18:**
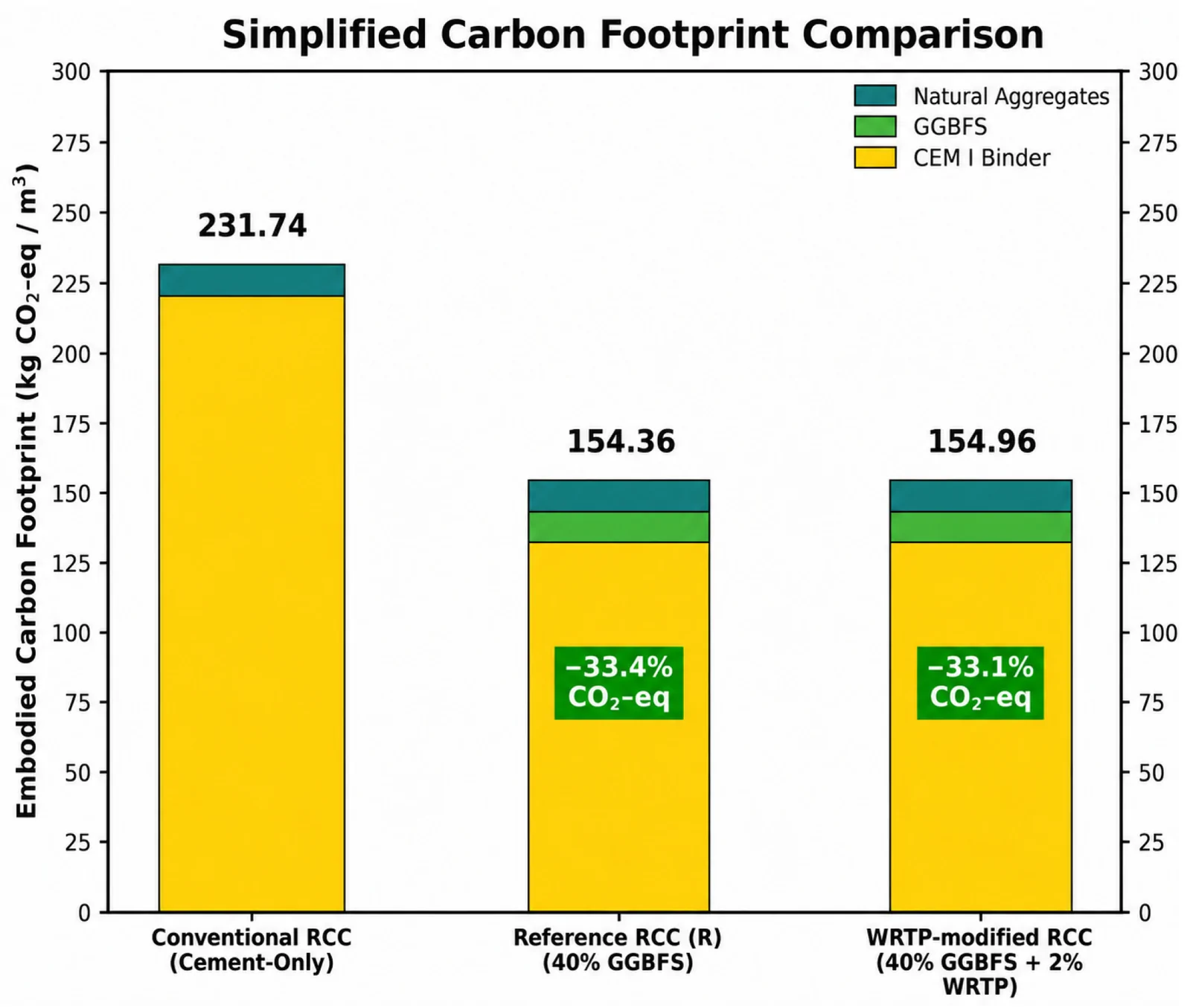
Simplified cradle-to-gate embodied-carbon comparison of the hypothetical cement-only RCC baseline, reference RCC, and 2% WRTP mixture.

As presented in Table 9, the simplified assessment indicates that replacing 40% of the Portland cement with GGBFS reduced the estimated material-related embodied carbon from approximately 231.74 kg CO_2_-eq/m^3^ for the hypothetical cement-only RCC baseline to 154.36 kg CO_2_-eq/m^3^ for the reference RCC mixture, corresponding to an estimated reduction of approximately 33.4%. The WRTP2 mixture exhibited an estimated embodied carbon of 154.96 kg CO_2_-eq/m^3^, corresponding to a reduction of approximately 33.1% relative to the hypothetical cement-only baseline. These results indicate that the principal reduction in estimated embodied carbon is associated with the 40% GGBFS binder substitution, whereas the incorporation of WRTP at the 2% replacement level results in only a minor change in the calculated embodied carbon. Accordingly, the environmental contribution of WRTP in the present mixtures is more appropriately interpreted in terms of waste utilization and material recovery rather than as an additional reduction in embodied carbon. The comparative contributions of the constituent materials to the estimated embodied carbon of the three mixtures are illustrated in Figure 18.

Beyond the embodied-carbon comparison, WRTP incorporation provides an additional waste-diversion benefit. The WRTP mixture incorporates 6.43 kg of waste tire-derived rubber per cubic meter of RCC. As an illustrative scale-up, a two-lane pavement section 1 km in length, 7.2 m in total width, and 0.20 m in thickness would require approximately 1440 m^3^ of RCC and would therefore incorporate approximately 9.26 tonnes of WRTP if the WRTP2 mixture were used. This calculation is presented solely to illustrate the potential scale of waste-material utilization and does not constitute a field-scale environmental assessment.

Because the present assessment relies on literature-derived ECFs rather than product-specific environmental declarations or primary life-cycle inventory data for the materials used in this study, the calculated values should be interpreted as a comparative screening-level embodied-carbon assessment rather than a complete LCA or product-specific carbon footprint. Actual environmental impacts may vary depending on material production processes, transportation distances, regional energy mixes, construction activities, service-life performance, and end-of-life scenarios.

## 4. Conclusions

This study evaluated the influence of replacing fine aggregate with WRTP at 0%, 2%, 4%, 8%, and 16% by volume on the fresh behavior, mechanical performance, physical and transport-related properties, and microstructure of RCC produced with a fixed binder system containing 40% GGBFS. In addition, a simplified embodied-carbon and waste-diversion assessment was conducted. The principal findings can be summarized as follows:

- WRTP incorporation reduced workability, with Ve-Be time increasing systematically; the 16% WRTP mixture showed an approximately 74% increase compared with the reference mixture.
- Density decreased with increasing WRTP content, consistent with the lower specific gravity of rubber and the higher likelihood of entrapped voids/discontinuities in compacted RCC.
- UPV decreased with WRTP incorporation; however, all mixtures remained within the “excellent” UPV classification range across the investigated curing ages.
- Compressive and flexural strengths reached their highest mean values at 2% WRTP. Beyond 2% WRTP, strengths generally decreased, with flexural strength remaining less sensitive than compressive strength—suggesting that compressive behavior is more strongly influenced by matrix continuity and void-related discontinuities, whereas flexural response may benefit from energy-dissipation and crack-resistance mechanisms.
- Splitting tensile strength showed numerical increases in all WRTP mixtures compared with the reference, suggesting that WRTP particles may contribute to crack-bridging and frictional energy dissipation at low-to-moderate replacement levels.
- Overall, WRTP incorporation tended to reduce capillary water absorption, particularly secondary absorption and initial absorption at higher replacement levels, suggesting reduced continuity of water-accessible pathways despite the observed density reductions.
- Water absorption and porosity showed their lowest mean values at 2% WRTP and generally increased at higher WRTP contents, with the 16% WRTP mixture exhibiting the highest values. The two properties were strongly related, with linear regression yielding R^2^ values of 0.996 and 0.970 at 28 and 90 days, respectively, indicating a strong association between pore and absorption behavior.
- Static modulus of elasticity decreased progressively with increasing WRTP content; however, the 2% WRTP mixture retained a stiffness level comparable to the reference, while higher replacement levels resulted in more pronounced reductions.
- SEM/EDS observations were consistent with the macroscopic trends: the 2% WRTP mixture exhibited less pronounced interfacial discontinuities, whereas more evident voids and discontinuities were observed at the rubber–paste interface in the 16% WRTP mixture. EDS showed higher local carbon signals in the WRTP-containing mixtures, consistent with rubber-rich regions in the analyzed areas; these observations should be regarded as qualitative and localized.
- Statistical analyses (one-way, two-way, and Welch ANOVA with appropriate post-hoc tests, α = 0.05) showed that the effect of WRTP varied across the evaluated properties. When the statistical results were considered together with the mechanical, physical, and transport-related performance, the 2% WRTP mixture provided the most favorable overall balance among the replacement levels investigated. At this replacement level, the static modulus of elasticity and 28-day porosity remained statistically comparable to those of the reference mixture, whereas the 90-day compressive strength was significantly higher and secondary capillary water absorption was significantly lower. In contrast, the increases observed in flexural and splitting tensile strengths were numerical and not statistically significant. From a practical engineering perspective, the 2% WRTP mixture reached a 90-day compressive strength of 37.79 MPa, exceeding the 27.6 MPa minimum value cited for RCC pavement surface layers.
- The simplified cradle-to-gate embodied-carbon assessment indicated that the 40% GGBFS binder substitution was the primary contributor to the estimated carbon reduction. Relative to the hypothetical cement-only RCC baseline, the reference and 2% WRTP mixtures showed reductions of approximately 33.4% and 33.1%, respectively. At the 2% replacement level, the contribution of WRTP was primarily associated with waste utilization, corresponding to 6.43 kg/m^3^ of waste tire-derived rubber, rather than with further embodied-carbon reduction. The assessment therefore provides a screening-level environmental comparison rather than a complete life-cycle assessment.

Overall, the findings emphasize that WRTP content should be carefully selected to achieve a favorable balance among the engineering properties of RCC, with the 2% WRTP mixture showing the most favorable overall performance among the investigated mixtures in terms of mechanical, physical, and transport-related properties. This performance was obtained for the specific materials, mixture proportions, and laboratory conditions considered in this study. Since the durability evaluation was limited to capillary water absorption, water absorption, and porosity, future studies should address pavement-related durability and long-term performance, including fatigue, abrasion, freeze–thaw resistance, drying shrinkage, skid resistance, chloride penetration, sulfate resistance, and carbonation.

Field-scale studies should further examine mixing and construction feasibility, field compaction, scale-up effects, and their implications for pavement performance. Comprehensive life cycle and techno-economic assessments should also consider product-specific environmental data, transportation, construction-stage impacts, service-life performance, and end-of-life scenarios. In addition, rubber surface treatment and mix-design refinements involving aggregate grading and compaction energy should be investigated to mitigate performance losses at higher WRTP contents.

## Figures and Tables

**Table 1:** Chemical properties of cement and GGBFS.

Chemical Properties	Composition	MgO	CaO	Fe_2_O_3_	SiO_2_	Al_2_O_3_	TiO_2_	Cl^−^	K_2_O	Na_2_O	SO_3_	LOI
GGBFS	(%)	5.85	35.61	1.52	39.14	13.34	1.44	-	0.33	0.76	0.19	0.01
Cement		1.39	63.15	3.15	19.25	4.27	-	0.041	0.56	0.44	3.61	2.70

**Table 2:** Physical characteristics of cement and GGBFS.

Physical Properties	Characteristics	Unit	Test Result
GGBFS	Specific surface area	cm^2^/g	5011
	Specific gravity	g/cm^3^	2.89
	Strength activity index (7th day)	%	55.7
	Strength activity index (28th day)	%	78.6
Cement	Specific surface area	cm^2^/g	3790
	Specific gravity	g/cm^3^	3.15

**Table 3:** D_10_, D_50_, and D_90_ values of the cement and GGBFS.

	D_10_	D_50_	D_90_
Cement	6.90 µm	19.1 µm	41.5 µm
GGBFS	1.02 µm	4.89 µm	12.5 µm

**Table 4:** Properties of the aggregates.

Fractions	Maximum Aggregate Size (mm)	Apparent Specific Gravity	Specific Gravity (OD)	Specific Gravity (SSD)	Water Absorption Capacity (%)	Fineness Modulus
0–3	3	2.85	2.37	2.54	7.17	1.8
0–5	5	2.72	2.29	2.45	7.00	2.6
5–12	12	2.80	2.69	2.73	1.49	3.9
12–22	22	2.72	2.63	2.66	1.14	5

**Table 5:** Particle size parameters and physical characteristics of WRTP.

	D_10_	D_50_	D_90_	Specific Surface Area	Particle Density
WRTP	66.7 µm	228 µm	451 µm	46.46 m^2^/kg	0.93 g/cm^3^

**Table 6:** Mixture proportions of the RCC mixtures.

Mixture	GGBFS (kg/m^3^)	Cement (kg/m^3^)	Waste Rubber Powder	Aggregate (kg/m^3^)				Water (kg/m^3^)
				0–3 mm	0–5 mm	5–12 mm	12–22 mm	
R	120	180	-	79.7	836.8	876.7	199.2	180.8
WRTP2	120	180	6.43	79.56	821.5	876.7	199.2	180.8
WRTP4	120	180	12.87	79.42	806.2	876.7	199.2	180.8
WRTP8	120	180	25.74	79.14	775.6	876.7	199.2	180.8
WRTP16	120	180	51.47	78.59	714.6	876.7	199.2	180.8

**Table 7:** Age-dependent second-order polynomial UPV–compressive strength regression models and cross-validation performance metrics.

Curing Age (Days)	Parabolic Regression Equation	R^2^	Adjusted R^2^	RMSE (MPa)	MAE (MPa)	Cross-Validated Q^2^	Cross-Validated RMSE (MPa)
7	CS = −584.86 + 231.18x − 22.22x^2^	0.83	0.8026	1.18	0.87	0.7642	1.39
28	CS = 2.62 − 7.99x + 2.38x^2^	0.80	0.7709	2.57	2.14	0.5337	3.96
90	CS = −692.91 + 260.03x − 23.22x^2^	0.88	0.8596	2.85	2.53	0.8122	3.56

**Table 8:** Initial and secondary capillary absorption coefficients of RCC mixtures.

Mixture	Initial Absorption (mm/√s) ± Standard Deviation	Secondary Absorption (mm/√s) ± Standard Deviation
Reference	0.00617 ± 0.00042	0.00127 ± 0.00009
WRTP2	0.00614 ± 0.00046	0.00086 ± 0.00011
WRTP4	0.00682 ± 0.00033	0.00114 ± 0.00014
WRTP8	0.00514 ± 0.00063	0.00094 ± 0.00003
WRTP16	0.00447 ± 0.00071	0.00107 ± 0.00019

**Table 9:** Simplified cradle-to-gate embodied carbon comparison and WRTP utilization of the hypothetical cement-only baseline, reference RCC, and 2% WRTP mixture.

Material/Parameter	ECF (kg CO_2_-eq/kg)	Hypothetical Cement-Only RCC	Reference	WRTP2
Cement (kg/m^3^)	0.735	300.00	180.00	180.00
GGBFS (kg/m^3^)	0.0901	0.00	120.00	120.00
0–3 mm aggregate (kg/m^3^)	0.007651	79.7	79.7	79.56
0–5 mm aggregate (kg/m^3^)	0.007651	836.8	836.8	821.5
5–12 mm aggregate (kg/m^3^)	0.0039	876.7	876.7	876.7
12–22 mm aggregate (kg/m^3^)	0.0039	199.2	199.2	199.2
WRTP (kg/m^3^)	0.113	0.00	0.00	6.43
Mixing water (kg/m^3^)	0.0002	180.8	180.8	180.8
Estimated embodied carbon (kg CO_2_-eq/m^3^)	-	231.74	154.36	154.96
Reduction relative to hypothetical baseline (%)	-	0.00	33.4	33.1
WRTP utilized (kg/m^3^)	-	0.00	0.00	6.43

## Data Availability

The original contributions presented in this study are included in the article. Further inquiries can be directed to the corresponding author.

## Appendix A. Descriptive Statistics and Post-Hoc Homogeneous Groupings

This appendix presents the descriptive statistics and post-hoc homogeneous groupings for the mechanical, non-destructive, physical, and transport-related properties of the RCC mixtures investigated in this study. The results are reported as mean values, standard deviations (SD), coefficients of variation (COV), and 95% confidence intervals (CI). Table A1 summarizes the mechanical properties and ultrasonic pulse velocity (UPV) results, whereas Table A2 presents the physical and transport-related properties. Post-hoc homogeneous groupings were determined at a significance level of α = 0.05. Tukey’s honestly significant difference (HSD) test was used for datasets satisfying the homogeneity-of-variance assumption, whereas the Games–Howell test was applied when this assumption was not satisfied. Within each property and curing age, mixtures sharing at least one letter are not statistically significantly different at α = 0.05.

**Table A1:** Mechanical and Non-Destructive Test Results.

Property	Mixture	Mean	SD	COV (%)	95% CI	Group
Compressive Strength (7 days) (MPa)	Reference	15.47	0.95	6.15	[13.11, 17.84]	A
	WRTP2	16.77	0.42	2.52	[15.72, 17.82]	A
	WRTP4	11.27	0.63	5.55	[9.72, 12.82]	B
	WRTP8	10.59	0.50	4.73	[9.34, 11.83]	BC
	WRTP16	9.66	0.16	1.66	[9.27, 10.06]	C
UPV (7-day) (km/s)	Reference	5.39	0.09	1.58	[5.18, 5.60]	A
	WRTP2	5.04	0.16	3.14	[4.65, 5.44]	B
	WRTP4	4.72	0.03	0.61	[4.64, 4.79]	C
	WRTP8	4.76	0.08	1.76	[4.55, 4.96]	C
	WRTP16	4.64	0.02	0.45	[4.58, 4.69]	C
Compressive Strength (28 days) (MPa)	Reference	26.57	0.57	2.15	[25.15, 27.98]	B
	WRTP2	28.22	0.32	1.14	[27.42, 29.02]	A
	WRTP4	17.23	0.31	1.80	[16.46, 17.99]	C
	WRTP8	15.97	0.55	3.44	[14.61, 17.34]	D
	WRTP16	14.16	0.27	1.94	[13.47, 14.84]	E
UPV (28 days) (km/s)	Reference	5.33	0.13	2.45	[5.01, 5.65]	A
	WRTP2	5.16	0.10	1.97	[4.91, 5.41]	A
	WRTP4	4.76	0.03	0.56	[4.69, 4.83]	B
	WRTP8	4.52	0.14	3.01	[4.18, 4.85]	B
	WRTP16	4.58	0.19	4.07	[4.11, 5.04]	B
Splitting Tensile Strength (MPa)	Reference	1.81	0.41	22.83	[0.78, 2.84]	A
	WRTP2	2.37	0.97	41.06	[0.82, 3.91]	A
	WRTP4	2.21	0.30	13.50	[1.47, 2.96]	A
	WRTP8	2.16	0.17	7.71	[1.75, 2.57]	A
	WRTP16	2.04	0.76	37.25	[0.83, 3.24]	A
Flexural Strength (MPa)	Reference	3.33	0.15	4.56	[2.95, 3.70]	A
	WRTP2	3.47	0.44	12.54	[2.39, 4.55]	A
	WRTP4	3.15	0.45	14.16	[−0.86, 7.15]	A
	WRTP8	3.03	0.21	6.78	[1.18, 4.87]	A
	WRTP16	2.97	0.17	5.66	[2.55, 3.39]	A
Static Modulus of Elasticity (GPa)	Reference	13.65	0.49	3.57	[12.44, 14.87]	A
	WRTP2	13.34	0.53	3.94	[12.03, 14.64]	AB
	WRTP4	12.23	0.45	3.71	[11.11, 13.36]	BC
	WRTP8	11.37	0.29	2.53	[8.79, 13.95]	CD
	WRTP16	10.25	0.13	1.23	[9.94, 10.56]	D
Compressive Strength (90 days) (MPa)	Reference	31.09	1.84	5.92	[26.52, 35.66]	B
	WRTP2	37.79	0.57	1.52	[36.36, 39.21]	A
	WRTP4	21.02	0.55	2.62	[19.65, 22.38]	C
	WRTP8	19.42	1.00	5.15	[16.93, 21.90]	C
	WRTP16	15.68	0.73	4.68	[13.86, 17.50]	D
UPV (90 days) (km/s)	Reference	5.42	0.06	1.12	[5.27, 5.57]	A
	WRTP2	5.35	0.02	0.29	[5.31, 5.38]	A
	WRTP4	4.78	0.06	1.15	[4.65, 4.92]	BC
	WRTP8	4.82	0.05	1.06	[4.70, 4.95]	B
	WRTP16	4.69	0.05	0.98	[4.58, 4.80]	C

**Table A2:** Physical and Transport-Related Properties.

Property	Mixture	Mean	SD	COV (%)	95% CI	Group
Density (28 days) (g/cm^3^)	Reference	2.568	0.041	1.60	[2.466, 2.670]	A
	WRTP2	2.509	0.005	0.20	[2.497, 2.522]	AB
	WRTP4	2.492	0.014	0.55	[2.458, 2.525]	BC
	WRTP8	2.432	0.013	0.52	[2.401, 2.463]	CD
	WRTP16	2.396	0.028	1.17	[2.326, 2.465]	D
Water Absorption (28 days) (%)	Reference	2.453	0.090	3.68	[2.229, 2.677]	C
	WRTP2	2.190	0.086	3.93	[1.977, 2.404]	C
	WRTP4	3.878	0.026	0.66	[3.814, 3.942]	A
	WRTP8	2.982	0.125	4.18	[2.673, 3.291]	B
	WRTP16	4.009	0.246	6.13	[3.399, 4.619]	A
Porosity (28 days) (%)	Reference	6.187	0.197	3.19	[5.697, 6.677]	C
	WRTP2	5.456	0.205	3.75	[4.947, 5.965]	C
	WRTP4	9.377	0.097	1.03	[9.137, 9.618]	A
	WRTP8	7.174	0.287	4.00	[6.461, 7.886]	B
	WRTP16	9.414	0.495	5.26	[8.185, 10.643]	A
Initial Capillary Absorption (mm/√s)	Reference	0.006174	0.000420	6.81	[0.005129, 0.007218]	AB
	WRTP2	0.006139	0.000465	7.57	[0.004985, 0.007293]	AB
	WRTP4	0.006824	0.000331	4.85	[0.003851, 0.009797]	A
	WRTP8	0.005137	0.000628	12.22	[0.003577, 0.006697]	BC
	WRTP16	0.004467	0.000712	15.93	[0.002699, 0.006234]	C
Secondary Capillary Absorption (mm/√s)	Reference	0.001265	0.000087	6.90	[0.001048, 0.001482]	A
	WRTP2	0.000858	0.000107	12.49	[0.000592, 0.001124]	B
	WRTP4	0.001140	0.000142	12.47	[−0.000137, 0.002416]	AB
	WRTP8	0.000935	0.000027	2.85	[0.000869, 0.001001]	B
	WRTP16	0.001073	0.000187	17.45	[0.000608, 0.001539]	AB
Density (90 days) (g/cm^3^)	Reference	2.512	0.021	0.82	[2.461, 2.563]	A
	WRTP2	2.439	0.011	0.43	[2.412, 2.465]	B
	WRTP4	2.430	0.010	0.39	[2.406, 2.453]	B
	WRTP8	2.394	0.041	1.73	[2.291, 2.497]	BC
	WRTP16	2.337	0.030	1.27	[2.263, 2.411]	C
Water Absorption (90 days) (%)	Reference	2.182	0.046	2.09	[2.068, 2.295]	A
	WRTP2	1.796	0.046	2.56	[1.682, 1.910]	B
	WRTP4	1.941	0.025	1.28	[1.879, 2.003]	B
	WRTP8	2.443	0.056	2.29	[2.304, 2.582]	C
	WRTP16	3.454	2.499	72.35	[−2.754, 9.661]	ABC
Porosity (90 days) (%)	Reference	5.430	0.142	2.61	[5.077, 5.782]	A
	WRTP2	4.405	0.109	2.48	[4.134, 4.677]	B
	WRTP4	4.693	0.037	0.79	[4.600, 4.786]	B
	WRTP8	5.810	0.122	2.10	[5.508, 6.113]	A
	WRTP16	7.710	5.098	66.13	[−4.955, 20.375]	AB

Note: Negative lower confidence limits, where present, result from the symmetric calculation of the 95% confidence interval in datasets with limited replicate measurements and/or high variability and do not represent physically negative property values.
